# Global trends in machine learning applications for single-cell transcriptomics research

**DOI:** 10.1186/s41065-025-00528-y

**Published:** 2025-08-16

**Authors:** Xinyu Liu, Zhen Zhang, Chao Tan, Yinquan Ai, Hao Liu, Yuan Li, Jin Yang, Yongyan Song

**Affiliations:** Clinical Medical College & Affiliated Hospital & College of Basic Medicine, Chengdu University, Chengdu, 610081 China

**Keywords:** Single-cell transcriptomics, Machine learning, Bibliometric analysis, Random forest, Deep learning

## Abstract

**Background:**

Single-cell RNA sequencing (scRNA-seq) has revolutionized cellular heterogeneity analysis by decoding gene expression profiles at individual cell level, while machine learning (ML) has emerged as core computational tool for clustering analysis, dimensionality reduction modeling and developmental trajectory inference in single-cell transcriptomics(SCT). Although 3,307 papers have been published in past two decades, there remains lack of bibliometric review comprehensively addressing methodological evolution, technical challenges and clinical translation pathways. This study aims to fill research gap through bibliometric and visual analysis, revealing technological evolution trends and future development directions.

**Methods:**

Using 3,307 publications from Web of Science Core Collection(WOSCC), we conducted bibliometric and visualization analysis through CiteSpace and VOSviewer to systematically review research trends, national/institutional contributions, keyword co-occurrence networks and co-citation relationships. Data screening strictly limited to English articles and reviews, excluding irrelevant document types, focusing on core application scenarios of ML in SCT.

**Results:**

China and United States dominated research output (combined 65%), with China leading in publication volume (54.8%) while US demonstrating academic influence through H-index 84 and 37,135 total citations. Research hotspots concentrated on random forest (RF) and deep learning models, showing transition from algorithm development to clinical applications (e.g., tumor immune microenvironment analysis). Chinese Academy of Sciences and Harvard University emerged as core collaboration hubs, with international cooperation network primarily featuring US-China collaboration. Keyword clustering revealed four themes: gene expression, immunotherapy, bioinformatics, and inflammation-related research. Technical bottlenecks included data heterogeneity, insufficient model interpretability and weak cross-dataset generalization capability.

**Conclusion:**

ML-scRNA-seq integration has advanced cellular heterogeneity analysis and precision medicine development. Future directions should optimize deep learning architectures, enhance model generalization capabilities, and promote technical translation through multi-omics and clinical data integration. Interdisciplinary collaboration represents key to overcoming current limitations (e.g., data standardization, algorithm interpretability), ultimately realizing deep integration between single-cell technologies and precision medicine.

**Supplementary Information:**

The online version contains supplementary material available at 10.1186/s41065-025-00528-y.

## Introduction

scRNA-seq is a method for analyzing gene expression at the single-cell level, revealing cellular heterogeneity and complex biological processes. Through high-dimensional measurements of gene expression dynamics at the single-cell level, this strategy has quickly grown into an efficient means for uncovering cellular heterogeneity [1–4]. The use of this technology in studies such as the characterization of transcriptional evolution during disease progression and the analysis of intercellular communication networks has fundamentally advanced our understanding of biological phenomena, including embryonic development, immune regulation, and tumor progression [5, 6]. However, the computational challenges associated with high dimensionality and complexity of single-cell data require the developers of analytical algorithms to combine advanced data mining and ML approaches to glean biologically informative knowledge from the data [7].

ML has revolutionized SCT by introducing efficient data analysis and interpretation methods. Key applications include clustering analysis (e.g., hierarchical, graph-based, and model-based clustering [8]) to identify cell types or states; dimensionality reduction (using Principal Component Analysis, t-Distributed Stochastic Neighbor Embedding, and Uniform Manifold Approximation and Projection [9]) for visualization and downstream analysis; trajectory inference (e.g., the deep learning model TIGON [10]) to reconstruct cellular developmental pathways; and cell type annotation (via combined deep learning and statistical approaches [11]), which significantly improves accuracy and efficiency. ML automates key analytical tasks, including identification of cellular properties, classification of cell types, and modeling of gene interactions [12], by combining traditional methods such as support vector machines (SVM) and RF with advanced architectures like autoencoders, graph-based neural networks, and transformer models [11]. These techniques enable rapid analysis of large-scale datasets while refining predictions of cellular behavior. The integration of machine learning and single-cell technologies has demonstrated significant value in cancer diagnosis, prediction of immunotherapy responses, and assessment of infectious disease severity. It helps identify key cellular subpopulations and immune biomarkers, advancing precision diagnostics and personalized treatment [13, 14]. This technological fusion is accelerating the intelligence and precision of clinical applications.Beyond accelerating research, ML-driven computational strategies enhance biological discovery by uncovering rare cell populations, improving disease diagnostics [15], and revealing previously inaccessible cell-state transitions through automated, high-dimensional pattern recognition [16]. This advances our understanding of tissue development, disease progression, and cellular dynamics, bridging computational innovation with biological insight.

In recent years, the interdisciplinary convergence between scRNA-seq and ML has evolved into a cutting-edge research frontier. Notably, Brendel’s research team [12] innovatively proposed an integrated deep learning-driven analytical methodology, establishing a multi-dimensional technological framework to realize systematic interpretation and modeling optimization of SCT data. Similarly, Brbic et al. [17] offer a concise overview of the latest ML approaches applied to single-cell sequencing data, detailing a range of computational frameworks and refined algorithmic optimizations. Those reviews can be overlapped by systematic approaches such as bibliometric analysis that offer a more systematic approach. Bibliometric approaches use analysis tools to deliver an objective record of research trends, cross-disciplinary linkages, and academic impact. Through the analysis of publication networks and citation relationships, bibliometric studies of this single-cell sequencing have reported, which can help in studying how machine learning is continuously utilized in SCT in a clear and recent manner. Rosales-Alvarez et al. [18] applied bibliometric techniques to map twelve years of single-cell sequencing studies, pinpointing key research hotspots and nascent trends in the field. Focusing on neurodegenerative diseases, Zhang et al. [19] conducted a visualization analysis of single-cell multiomics research, systematically summarizing current research progress while identifying key trends and emerging frontiers in the field.

The rapid expansion of scientific literature presents both opportunities and challenges for researchers. Without effective reading and analytical strategies, navigating the vast amount of available research can become overwhelming. Bibliometric analysis has emerged as a valuable method for understanding research field dynamics, providing insights into publication patterns, key research topics, and collaborative networks, while offering a quantitative and systematic approach to evaluate research progress and impact within a given domain [20, 21]. By employing bibliometrics and visualization techniques including CiteSpace and VOSviewer, researchers can efficiently monitor publication trends [22, 23], identify pivotal studies, recognize influential authors and leading research institutions, and focus on high-impact literature [24]. This organized approach simplifies the research process, helping scholars access reliable, high-quality sources while keeping up with the latest developments in the field [25]. So far, there hasn’t been a bibliometric analysis conducted on the fields of ML and SCT. Aiming to fill this gap, this bibliometric analysis offers a comprehensive and high-level summary of the present status of ML and SCT, highlighting its potential to revolutionize biological understanding and change medical practices.

We present a broad-scale bibliometric and visualization analysis of SCT research driven by ML in this study. We perform a systematic investigation on the trends of publication, global contributions, institutional collaborations, journal distributions, and co-citation networks. This study adopts keyword co-occurrence analysis and hot spot analysis technology to systematically reveal the evolutionary pathways and disciplinary development paradigms in this field. Through systematic literature survey, this work innovatively constructs a panoramic view of ML applications in SCT technology integration, with specific focus on: (1) providing an organized summary of ML methods, which may be particularly useful for eager researchers and practitioners intending to adopt computational approaches in single-cell data analysis, and for those who are more interested in following the trends in the field of computational biology targeting single-cell biology; and (2) highlighting major progress, challenges and opportunities in the intersection of ML and single-cell biology. We complement classical review approaches with bibliometric analyses, which provide a macro view of the contemporary landscape and future directions of scRNA-seq and ML.

## Materials and methods

### Data sources and search strategies

The WoSCC is the primary database for publications using ML methods for SCT studies [26]. It is the most trustworthy, complete, and prominent citation database for bibliometric research [27–29].

CiteSpace serves as a visualization platform for citation networks, purpose-built to unveil emerging trends in scholarly literature [30]. The fundamental purpose of knowledge graph construction technology lies in dynamically capturing the complete trajectory of the knowledge production life cycle [31]. Using visualization tools, a diagram can be created that represents how knowledge is gathered, arranged, and distributed. We used CiteSpace for analyzing countries, references, keywords, and journals [32]. We employed citation burst detection to uncover newly emerging studies and pivotal terms, and utilized a dual-map overlay of journals to illustrate the interconnections among the cited publications. Introduction of a co-cited literature network visualizations perspective time-zone perspective in the range of co-cited literature network visualizations, this is a plus to temporal evolution of research.

VOSviewer is a software tool used to create and visualize bibliometric maps [33]. It provides interface summary tables and performs in-depth bibliometric analysis [34]. Each node on the VOSviewer map corresponds to a specific parameter, such as countries/regions, institutions, or authors [35]. The size of each node reflects its relative importance and is determined by weighted metrics such as publication count, occurrence frequency and citation count. The color of the nodes and the clusters to which they belong is determined by their grouping [36]. Links between nodes are represented by lines, with the link strength evaluated using the Total Link Strength (TLS) index, which reflects the overall collaboration and co-citation link strength for countries or institutions.

Co-authorship analysis investigates the connections between authors, institutions, and countries, while co-occurrence analysis is a quantitative method that reveals the relationships between various elements. We applied statistical citation analysis to evaluate the impact of papers with high citation rates. For this study, we compiled essential metadata—author names, affiliated institutions, geographic locations, journal titles, keyword terms and reference lists.

This study carries out literature collection operations through the Topic Search (TS) methodology within the WOSCC database, integrating standardized subject terms from the MeSH thesaurus to complete the retrieval logic design. The structured retrieval scheme is formulated as follows: TS=(“Single-Cell Gene Expression Analysis” OR “Single-Cell Gene Expression Profiling” OR “Single-Cell Transcriptome Analysis” OR “Analyses, Single-Cell Transcriptome” OR “Analysis, Single-Cell Transcriptome” OR “Single-Cell Transcriptome Analyses” OR “Transcriptome Analyses, Single-Cell” OR “Transcriptome Analysis, Single-Cell” OR “Single Cell Gene Expression Analysis” OR “Single Cell Gene Expression Profiling” OR “Single Cell Transcriptome Analysis” OR “Single-Cell RNA-Seq” OR “RNA-Seq, Single-Cell” OR “Single Cell RNA Seq” OR “Single-Cell RNA Seq” OR “RNA Seq, Single-Cell” OR “Seq, Single-Cell RNA” ) AND TS=(“Machine learning” OR “Naive Bayes” OR “Decision trees” OR “Random Forest” OR “Support vector machines” OR “Gradient boost-ing decision tree” OR “Adaptive boosting” OR “Extreme gra-dient boosting” OR “Light gradient boosting machine” OR “Categorical boosting” OR “Generalized additive model” OR “Artificial neural networks” OR “Data Mining”” OR “Deep learning” OR “Learning, Machine” OR “Transfer Learning” OR “Learning, Transfer” OR “Machine Learning”).Inclusion criteria for the literature: studies published between 1997 and 2024, focusing on English-language publications, and limited to articles and review articles. Exclusion criteria: literature published in 2025, non-English publications, and materials other than articles and review articles.The study reported following the Preferred Reporting Items for Systematic Reviews (PRISMA) [37] guidelines (eTables 1 and 2 in the Supplement).

### Visualized analysis

The primary software tools used in this study include Excel2021, CiteSpace(6.2.R1), the R package bibliometrix(4.4.2), Scimago(1.0.48), and VOSviewer(1.6.20) [38]. This study constructs core methodological support based on the CiteSpace bibliometric platform, implementing systematic processing and knowledge mining operations on the standardized dataset [39] from WOSCC. The analytical framework effectively supports visual reconstruction of knowledge networks, focusing on three major dimensions: co-authorship network mapping, keyword co-occurrence clustering analysis, and literature co-citation network deconstruction, to systematically deconstruct the core methodological paradigms of bibliometrics [40].

This research relies on Microsoft Excel 2021 to conduct metrological analysis on literature output volume and citation frequency within relevant domains, simultaneously accomplishing the construction of visual mapping atlases. R Studio, a command-based software requiring input of relevant code for literature analysis, was utilized with the “bibliometrix” package [41]. Scimago was employed for geographical visualization in collaboration with VOSviewer [42].

**Fig. 1:**
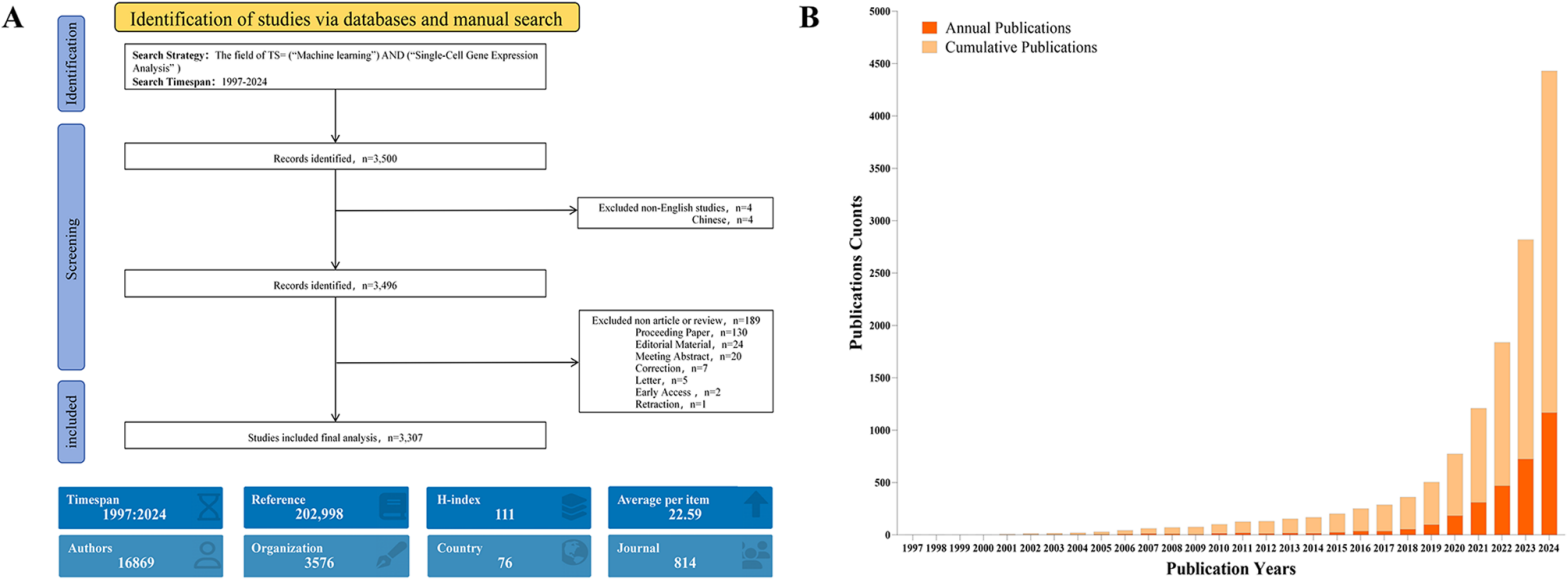
**(A)** Flowchart of Literature Search Strategy and Selection Process for Machine Learning and Single-Cell Transcriptomics. **(B)** Annual and Cumulative Publication Output in Machine Learning and Single-Cell Transcriptomics Research (1997–2024)

## Results

### Global trends in publication outputs and citations

Under rigorous literature selection criteria, this research ultimately included 3,307 English-language articles published from 1997 to 2024. Figure 1B systematically visualizes the academic productivity trends and scholarly impact evolution of machine learning applications in SCT through bibliometric mapping. The data indicates sluggish progress during the early 21st century, followed by a notable growth inflection point after 2019, with annual publication numbers sharply rising to approximately 1,200 papers in the 2023–2024 period. The main reason is that transcriptomics was selected as the“Method of the Year 2020,”which drew more researchers’attention to this field. A critical observation reveals that studies employing machine learning methodologies constituted 50% of total outputs between 2019 and 2024, while annual publication counts remained below 1,000 before 2018, with near-zero research activity recorded in 1997.

### Distribution of countries/regions

**Fig. 2:**
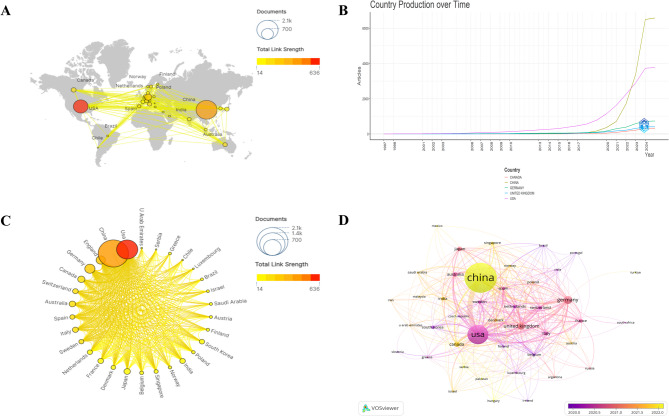
**(A)** Geographical distribution map based on the total publications of different countries/regions. **(B)** Temporal trends in production outputs of the top 5 countries/regions. **(C)** Visualization map of international collaborations by countries/regions. **(D)** The countries/regions’ citation network visualization map was generated by using a VOS viewer. The thickness of the lines reflects the citation strength

Relevant academic achievements have covered 76 geographical units. Geographical visualization analysis reveals (Fig. 2A) that SCT technology research with deeply integrated ML demonstrates significant geographical agglomeration characteristics, these publications are concentrated in three principal regional clusters: Asia, North America and Europe. Table 1 details the ten leading countries or regions contributing ML-related SCT studies, and Fig. 2B illustrates the publication trajectories of the top five from 1997 to 2024. Based on global scientific research output analysis (Fig. 2B), China demonstrates remarkable growth momentum in machine learning-driven SCT, ranking first worldwide in annual paper publication growth rate. In terms of academic output volume, Chinese scholars contributed 54.8% of research achievements in this field (1,809 papers), far surpassing the United States (20.5%, 677 papers) and Germany (3.6%, 118 papers). Notably, while China leads in total paper quantity, the United States maintains academic influence superiority, with cumulative citations reaching 37,135 times and H-index scoring 84, both ranking first globally. China follows with 18,434 citations.This may be related to limitations in literature access channels, as well as researchers’fields of study and language preferences.

Regarding scientific collaboration patterns, the United Kingdom, Australia, France, and Germany exhibit high levels of research internationalization, reflected by their significantly higher proportions of Multinational Cooperation Papers (MCP). International research collaboration network visualization (Fig. 2C) reveals that the United States has established the most extensive cooperation network, with China being its primary collaborative partner. European countries form regional collaboration clusters, particularly showing dense multinational cooperation among Germany, the United Kingdom, and France.The global collaboration network map constructed by VOSviewer (Fig. 2D, minimum publication threshold set at 5 papers) includes 43 countries/regions. The United States dominates the Total Link Strength (TLS = 661) metric measuring international collaboration intensity, followed by China (TLS = 423), England (TLS = 296), Germany (TLS = 286), and Australia (TLS = 157). This data reveals differentiated patterns of collaborative innovation in SCT research, while confirming China’s dual strengths in both academic output scale and sustainable development potential within this field.

**Table 1:** Top 10 most productive countries/regions in machine Learning-Based Single-Cell transcriptomics

Rank	Country	Count	Percentage	H-index	SCP	MCP	MCP-Ratio	TLS	TC	Average citation per paper
1	China	1809	54.8	57	1569	240	0.133	423	18,434	10.01
2	USA	677	20.5	84	498	179	0.264	661	37,135	43.79
3	Germany	118	3.6	37	67	51	0.432	286	9590	49.18
4	England	78	2.4	39	35	43	0.551	296	7245	47.98
5	Canada	62	1.9	29	32	30	0.484	157	4846	52.11
6	Australia	54	1.6	21	22	32	0.593	124	3011	36.28
7	India	51	1.5	14	31	20	0.392	58	542	8.74
8	Japan	50	1.5	20	38	12	0.24	82	1580	19.27
9	Korea	41	1.2	15	30	11	0.268	42	2251	43.29
10	France	35	1.1	19	21	14	0.4	108	2352	40.55

Note(s): H-index: The H-index of the Country, which measures both the productivity and citation impact of the publications. SCP: Single Country publications. MCP: Multiple Country Publications. MCP-Ratio: Proportion of Multiple Country Publications. TLS: Total Link Strength. The total strength of connections between countries and all other countries. TC: Total citations. Average Citations: The average number of citations per publication

### Contributions of institutions

The application papers in SCT based on ML include contributions from 3,576 institutions. Table 2 lists the top ten publishing institutions. The majority of research institutions involved in scientific research are from USA and China. Most of the articles are from the Chinese Academy of Sciences, Harvard University, Central South University, and Shanghai Jiao Tong.

**Fig. 3:**
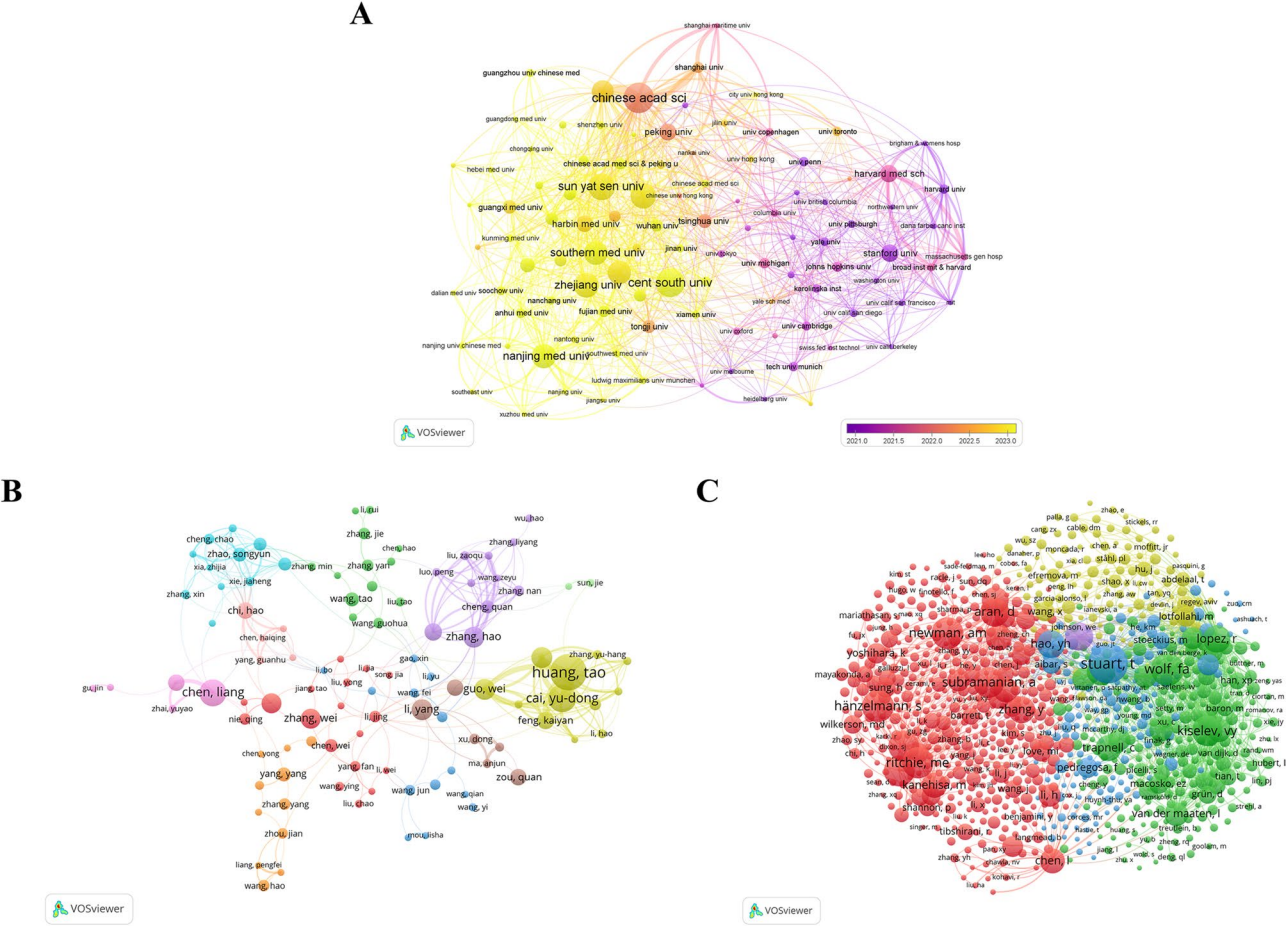
**(A)** The institutions’ collaboration network visualization map generated by VOSviewer software. **(B)** Visualization analysis of author collaboration networks in VOSviewer.This figure displays authors with three or more publications. Nodes of different colors represent authors from distinct clusters, and the node size corresponds to the frequency of their appearances (i.e., publication count). **(C)** Visualization analysis of citation-based collaboration networks in VOSviewer.The node size reflects the frequency of their appearances (i.e., citation count)

Analysis of the data in Table 2 indicates that the top ten global research universities collectively contributed 930 scholarly outputs in this domain, representing 28.19% of total academic production. Harvard University dominates in dual impact metrics, specifically achieving an average of 32.6 citations per publication while maintaining an H-index of 78, thereby solidifying its position as the field’s academic leader. Analysis of academic collaboration networks demonstrates that these institutions have established interdisciplinary innovation clusters through sustained intellectual exchanges. The network visualization includes institutions that have published no fewer than 16 papers. As illustrated in Fig. 3A, a total of 103 nodes are present in the map. Notably, the Chinese Academy of Sciences (TLS = 167), the University of Chinese Academy of Sciences (TLS = 139), and Harvard University (TLS = 121) demonstrate the strongest collaborative links, as indicated by their high TLS values.

**Table 2:** The top 10 productive institutions ranked by the numbers of publications

Rank	Institutions	Count	Country	H-index	TLS	TC	Centrality	Average citation per paper
1	Chinese Academy of Sciences	140	China	20	167	1663	0.21	11.88
2	Harvard University	111	USA	32	121	6992	0.30	62.99
3	Central South University	92	China	14	70	795	0.08	8.64
4	Shanghai Jiao Tong University	92	China	13	115	679	0.11	7.38
5	Sun Yat-sen University	89	China	14	110	1043	0.09	11.72
6	University of Chinese Academy of Sciences	84	China	18	139	803	0.19	9.56
7	Nanjing Medical University	83	China	15	115	910	0.07	10.96
8	Fudan University	80	China	16	69	762	0.07	9.53
9	Southern Medical University	80	China	15	107	703	0.08	8.79
10	Zhejiang University	79	China	16	62	1083	0.09	13.71

Note(s): H-index: The H-index of the Institution, which measures both the productivity and citation impact of the publications. TLS: Total Link Strength. The total strength of connections between institutions and all other institutions. TC: Total citations. Average Citations: The average number of citations per publication.Centrality: A quantitative metric for assessing the importance of institutional nodes

### Author and co-cited author analysis

According to bibliometric theory, when two or more researchers’ academic achievements are cited together in one or multiple follow-up research papers, a co-citation connection is formed. The closeness of such academic associations can be quantitatively assessed by calculating co-citation frequency, specifically manifested as the synchronized citation frequency of different researcher combinations within citation networks. A higher co-citation frequency between these authors suggests a stronger academic relationship. Co-citation analysis using ML methods on SCT research can not only reveal the current state of development and scientific structure, but also identify frontiers and provide scientific evaluation, supporting macro-level science and technology decision-making. Table 3 presents detailed information about the top 10 authors in the field of SCT based on ML. This includes the number of publications, co-citation rates, affiliated institutions, and TLS. Huang, Tao from Peking University ranks first with 17 publications and the highest TLS of 117. Following closely is Cai, Yu-Dong from Shanghai University, with 15 publications and a TLS of 103. Zou, Quan from the University of Electronic Science and Technology of China has 13 publications, but a much lower TLS of just 2. In VOSviewer, the collaboration relationships between authors related to publications in SCT research based on ML are shown in Fig. 3B. The larger nodes of Huang, Tao and Cai, Yu-Dong indicate their significant contributions to author collaboration relationships. Huang, Tao collaborates with Zhang, Hao, Zhang, Jie, Li, Rui, and Li, Yang, while Cai, Yu-Dong has close collaborations with Li, Hao, Xu, Dong, Ma, Anjun, and Zou, Quan. The co-cited author network map (Fig. 3C) shows that Stuart, T (Genome Institute of Singapore, cited 542 times) is the most co-cited author, followed by Wolf, FA (Computational Health Center, Helmholtz Center Munich, cited 384 times).

**Table 3:** Top 10 authors in terms of publications counts

Rank	Author	Count	Institutions	TLS
1	Huang, Tao	17	Peking University	117
2	Cai, Yu-Dong	15	Shanghai University	103
3	Zou, Quan	13	University of Electronic Science and Technology of China	2
4	Deng, Minghua	13	Peking University	20
5	Yang, Fantang	12	Fermi National Accelerator Laboratory	10
6	Zhao, Songyun	12	wuxi people’s hospital	37
7	Chi, Hao	11	Southwest Medical University	45
8	Guo, Wei	11	Shanghai Jiao Tong University	70
9	Li, Zhandong	10	Northeast Normal University	43
10	Wong, Ka-chun	9	City University of Hong Kong	103

Note(s): TLS: Total Link Strength. The total strength of connections between an author and all other authors

### Analysis of journal distribution and research areas

By using bibliometric analysis of journals, we can apply ML methods related to these fields to identify key journals in SCT. The impact factor, journal citation reports (JCR) rankings, H-index, and total citations are included in Table 4, which lists the top 10 journals by publication volume in this field. Frontiers in Immunology (187 papers), Briefings in Bioinformatics (139 papers), and Frontiers in Genetics (97 papers) are the top three journals with the highest number of articles published on ML-based SCT. Switzerland accounts for 30% (3/10) of the top 10 journals, while England dominates with 70% (7/10).The main point lies in the fluency of the language, as English is the most widely used language in the world. The top 10 most active journals published 880 articles, representing 26.67% of all publications. Among the journals that published more than 40 articles, Nature Communications has the highest H-index (H = 33), followed by Briefings in Bioinformatics and Genome Biology, both with an H-index of 22. These are followed by Bioinformatics (H = 20) and Frontiers in Immunology (H = 19). Among the top 10 journals, 8 belong to the Q1 quartile (the top 25% by impact factor), and the remaining 2 belong to the Q2 quartile (the 25–50% range). Nature Communications stands out with the highest total citations (6383), the highest impact factor (IF = 14.7), and the highest H-index (33). These findings suggest that journals like Nature Communications play a significant role in advancing research in this field.

**Fig. 4:**
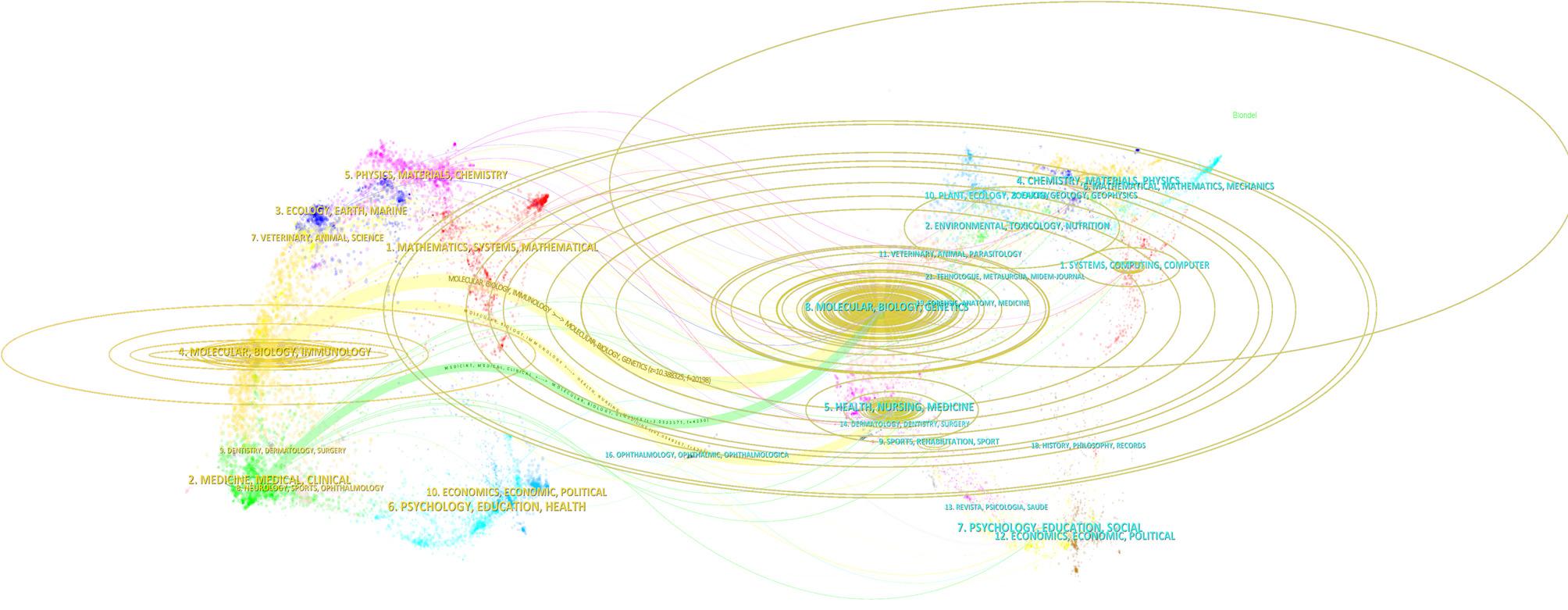
The dual-map overlay of journals. The labels on the left represent citing journals, the labels on the right represent cited journal, and colored paths indicate citation relationships

To analyze the relationships between academic citations and co-citations, we used CiteSpace’s dual-map overlay of academic journals (Fig. 4), which displays the distribution of journal topics. The map illustrates various research domains covered by the journals, with citing sources placed on the left and the corresponding cited journals on the right. Citation paths are represented by lines of different colors, with each line starting from a citing map and eventually reaching the target journal. The green path indicates that journals in the health and nursing fields may cite research from psychology and education. The orange path suggests that journals in molecular biology and immunology are often cited by journals in medicine and clinical research. The width of the connecting paths is closely related to the citation frequency on the z-score scale. The “Molecular, Biology, Immunology” cluster, located in the upper-left of the map, primarily cites literature from “Molecular, Biology, Genetics” and “Health, Nursing, Medicine”, reflecting its strong foundation in life and clinical sciences. The “Medicine, Medical, Clinical” area in the lower-left draws on sources from “Environmental, Toxicology, Nutrition”, “Molecular Biology”, and “Health-related disciplines”, indicating integration of environmental and nutritional factors into clinical research. Meanwhile, “Physics, Materials, Chemistry” and “Psychology, Education, Health” frequently reference computer science, engineering, and social sciences, showing increasing interdisciplinarity. Notably, “Sports, Rehabilitation, Exercise” connects closely with “Chemistry, Materials, Physics”, highlighting active knowledge exchange in areas such as biomechanics and rehabilitation technologies.From the map, research in SCT using ML methods follows an interdisciplinary development pattern.

**Table 4:** Top 10 journals in machine Learning-Based Single-Cell transcriptomics

Rank	Journal	Country	Count	IF	JCR	H-index	TC	Percentage
1	Frontiers In Immunology	Switzerland	187	5.7	Q1	19	1405	5.668
2	Briefings In Bioinformatics	England	139	6.8	Q1	22	1848	4.213
3	Frontiers In Genetics	Switzerland	97	2.8	Q2	16	833	2.940
4	Scientific Reports	England	97	3.8	Q1	12	805	2.940
5	Nature Communications	England	87	14.7	Q1	33	6383	2.637
6	Bioinformatics	England	85	4.4	Q1	20	1175	2.576
7	Bmc Bioinformatics	England	52	2.9	Q2	16	1428	1.576
8	International Journal Of Molecular Sciences	Switzerland	48	4.9	Q1	9	409	1.454
9	Genome Biology	England	44	10.1	Q1	22	6029	1.333
10	Heliyon	England	44	3.4	Q1	4	59	1.333

Note(s): IF: Impact Factor, indicating the average number of citations to recent articles published in the journal. JCR: The quartile ranking of the journal in the Journal Citation Reports, indicating the journal’s ranking relative to others in the same field (Q1: top 25%, Q2: 25%-50%, Q3: 50%-75%, Q4: bottom 25%). H-index: The H-index of the Journal, which measures both the productivity and citation impact of the publications. TC: Total citations

### Analysis of top co-cited references

**Fig. 5:**
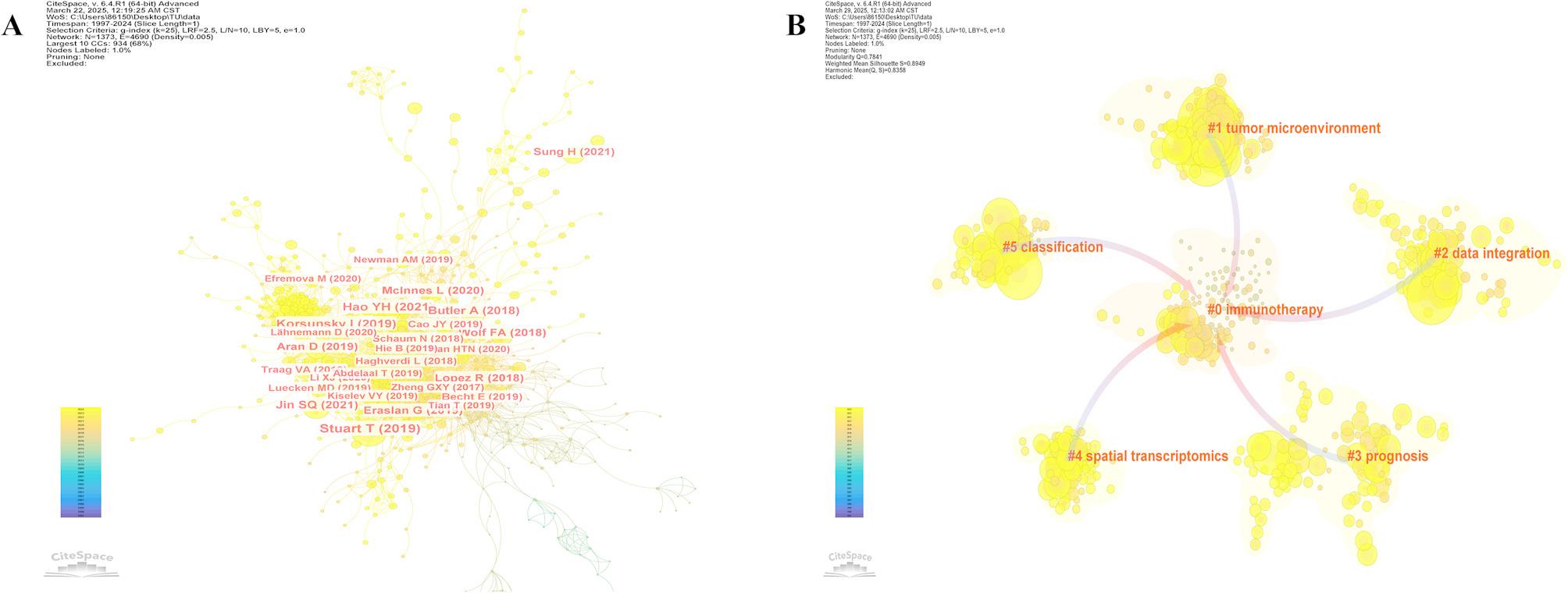
**(A)** Citespace visualization timeline view of co-citation references. The time evolution is indicated with different colored lines, and the nodes on the lines indicate the references cited. **(B)** Clustering analysis of articles co-citation.The parameters were set as follows: Time slice (1997–2024), year per slice (1), selection criteria (K = 5)

In our investigation, a total of 131,786 references were cited. Table 5 presents the top ten most cited papers in the field of ML-based SCT. Tim Stuart [1] received the highest number of citations, with 434 citations, followed by Sonja Hänzelmann [43], who was cited 372 times. Among the journals where the cited articles were published, the top three journals with the highest impact factors are Cell (IF = 45.6), Nature Methods (IF = 36.1), and Nature Biotechnology (IF = 33.1), which have the greatest influence in the field. Figure 5A displays the co-citation analysis of literature related to ML and SCT, generated by CiteSpace. The results show that the most cited article is by Tim Stuart (2019) [1], which becomes a key node in the co-citation network, acting as a bridge or hub within the entire network. Stuart [1] and Korsunsky [44] enable cross-platform single-cell data integration and have played a crucial role in constructing the human immune atlas. WGCNA has shifted research from single-gene analysis to modular co-expression networks and has been applied to disease mechanisms such as Alzheimer’s disease [45]. GSVA and GSEA have advanced expression analysis from the gene level to pathway activity, aiding in breast cancer subtype identification and precision treatment [46]. SCANPY has enhanced large-scale single-cell data processing in the Python environment and has been widely used in COVID-19 immune studies [47]. clusterProfiler [48] has optimized the standardization and visualization of enrichment analyses, CIBERSORT enables immune cell composition estimation from bulk RNA-seq data, and Ritchie [49] along with its voom method has established the standard pipeline for differential expression analysis. Together, these methods have shaped the core framework of modern bioinformatics analysis.

Figure 5B Cluster analysis shows that research on ML and SCT can be categorized into six distinct themes, covering areas such as mechanisms, treatment, and prediction, involving the intersection of biology, medicine, and data science. It is worth noting that the focus of research has shifted over time. In the early stages, research predominantly concentrated on particular therapeutic strategies, such as immunotherapy. However, in recent years, the attention has progressively expanded toward more comprehensive technologies and diverse methodological frameworks. Key topics have evolved from #0 Immunotherapy to #1 Tumor microenvironment, #2 Data integration, #3 Prognosis, #4 Spatial transcriptomics, and #5 Classification.

Cluster #1: Tumor Microenvironment (TME) This cluster primarily investigates the interactions between immune cells and stromal cells, focusing on their roles in treatment resistance. Random forest models are employed to identify various subtypes of exhausted T cells [50]. Cluster #2: Data Integration This cluster synthesizes multiple data types, including scRNA-seq and ATAC-seq. Generative adversarial networks (GANs) and Transformer models are utilized to mitigate batch effects and harmonize data from disparate platforms [1]. Cluster #3: Prognosis Survival prediction models are developed based on single-cell data, with the identification of biomarkers such as immune microenvironment scores to enhance prognostic accuracy [51]. Cluster #4: Spatial Transcriptomics Graph neural networks and TLS density scores are applied to correlate cellular spatial locations with their functions, thereby facilitating more precise immunotherapy strategies [52]. Cluster #5: Classification Support vector machines and deep forest algorithms are employed for precise cell classification, effectively identifying rare cell types such as tumor stem cells [53].

Case studies demonstrate that bibliometric clustering serves as an effective method for mapping the evolving research paradigms in single-cell biology. In the field of immunotherapy (Cluster #0), the shift towards exploring the mechanisms of the tumor microenvironment (TME) (Cluster #1) marks a critical change (Fig. 5B). This transition was primarily driven by scRNA-seq, which uncovered the functional suppression of dendritic cells (DCs) and the presence of heterogeneous exhausted T cell subsets, such as LAG3+/TIM3+, within the TME. Random forest modeling was employed to quantify the spatial interactions between DCs and T cells [54], which directly informed the development of a combinatorial therapy utilizing a PD-1 antibody and DC activator. This approach led to a 40% increase in clinical response rates. Concurrently, spatial transcriptomics (Cluster #4) emerged as a distinct technological paradigm (Fig. 5B). Spatial mapping identified TLS, where the proximity of DCs and CD8 + T cells served as a predictor of treatment efficacy. Additionally, cancer-associated fibroblasts (CAFs) formed CXCL12-mediated immune exclusion zones. Graph neural networks (GNNs) were used to translate these insights into a “TLS density score” biomarker [55], facilitating patient stratification for DC vaccines and advancing spatially targeted therapies.

**Table 5:** Top 10 co-cited references in machine Learning-Based Single-Cell transcriptomics

Title	First author	Journal	IF	JCR	Year	Citations
Comprehensive Integration of Single-Cell Data	Tim Stuart	Cell	45.6	Q1	2019	434
GSVA: gene set variation analysis for microarray and RNA-seq data	Sonja Hänzelmann	BMC Bioinformatics	2.9	Q2	2013	372
limma powers differential expression analyses for RNA-sequencing and microarray studies	Matthew E Ritchie	Nucleic Acids Res	16.7	Q1	2015	362
WGCNA: an R package for weighted correlation network analysis	Peter Langfelder	BMC Bioinformatics	2.9	Q2	2008	330
SCANPY: large-scale single-cell gene expression data analysis	F Alexander Wolf	Genome Biol	10.1	Q1	2018	316
Gene set enrichment analysis: a knowledge-based approach for interpreting genome-wide expression profiles	Aravind Subramanian	Proc Natl Acad Sci USA	9.4	Q1	2005	311
clusterProfiler: an R package for comparing biological themes among gene clusters	Guangchuang Yu	OMICS	2.2	Q3	2012	305
Integrating single-cell transcriptomic data across different conditions, technologies, and species	Andrew Butler	Nat Biotechnol	33.1	Q1	2018	303
Fast, sensitive and accurate integration of single-cell data with Harmony	Ilya Korsunsky	Nat Methods	36.1	Q1	2019	286
Robust enumeration of cell subsets from tissue expression profiles	Aaron M Newman	Nat Methods	36.1	Q1	2015	273

Note(s): IF: Impact Factor, indicating the average number of citations to recent articles published in the journal. JCR: The quartile ranking of the journal in the Journal Citation Reports, indicating the journal’s ranking relative to others in the same field (Q1: top 25%, Q2: 25%-50%, Q3: 50%-75%, Q4: bottom 25%)

**Fig. 6:**
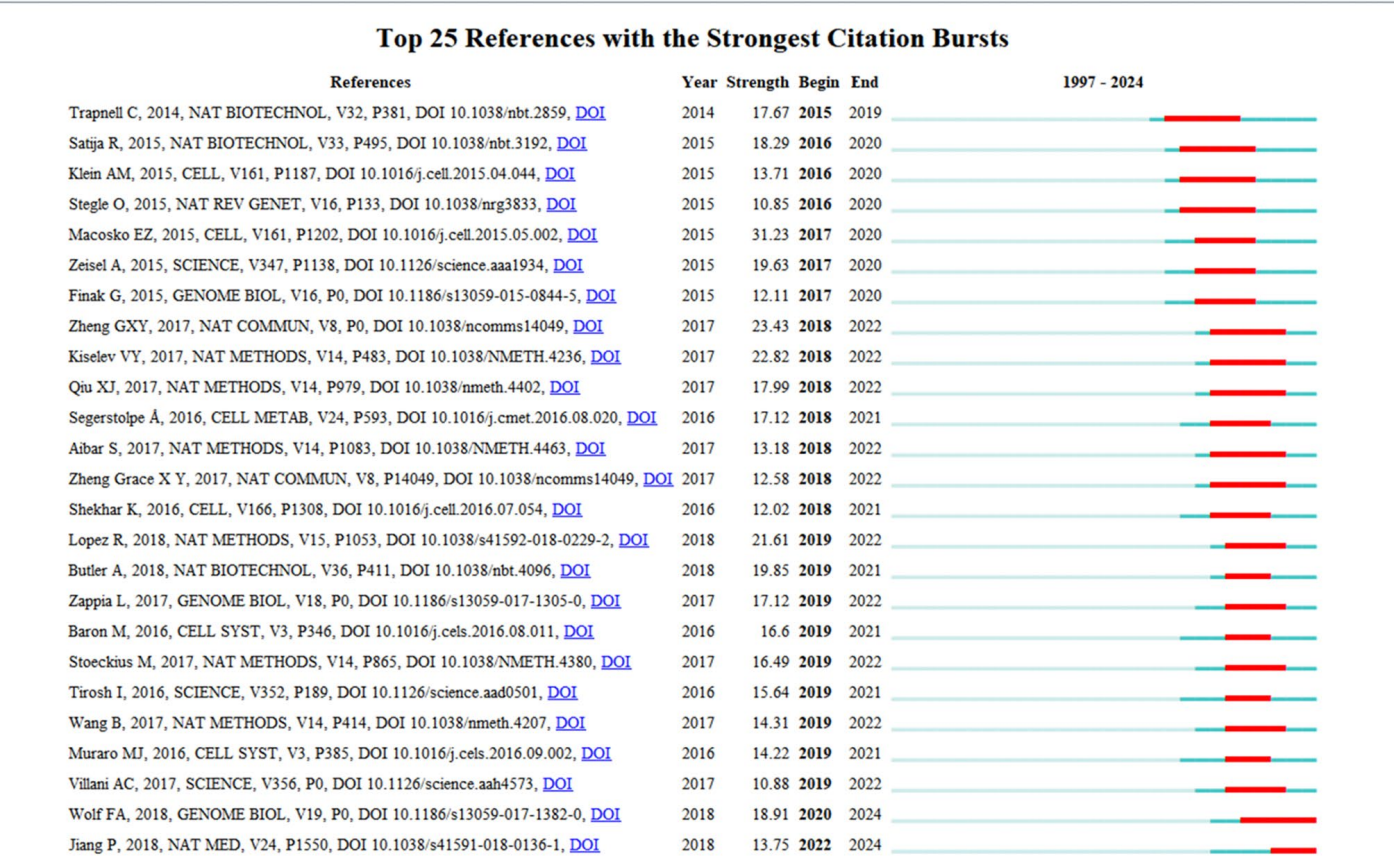
Top 20 literatures with the strongest citation bursts analysis. The red areas in the graph represented the period when the number of citations for each article surged

Figure 6 centrally presents 25 core references demonstrating the most prominent citation surge effects. Since 2017, this discipline has shown a quantum leap in citation volume, with multiple co-citation-characterized references maintaining high academic attention throughout subsequent research cycles. This dynamic evolution pattern of citations not only reflects the migration trajectory of research hotspots but also reveals the sustained influence of foundational achievements within the field. This underscores that ML-driven SCT continues to be prominent research hotspots. Notably, the paper by Jiang P [56], which integrates clinical data for personalized medicine analysis, and the study by Wolf FA [7], which applies ML methods to analyze scRNA-seq data, have citation bursts extending into 2024, reaffirming their relevance as current research frontiers.

### Keywords analysis of research hotspots

**Fig. 7:**
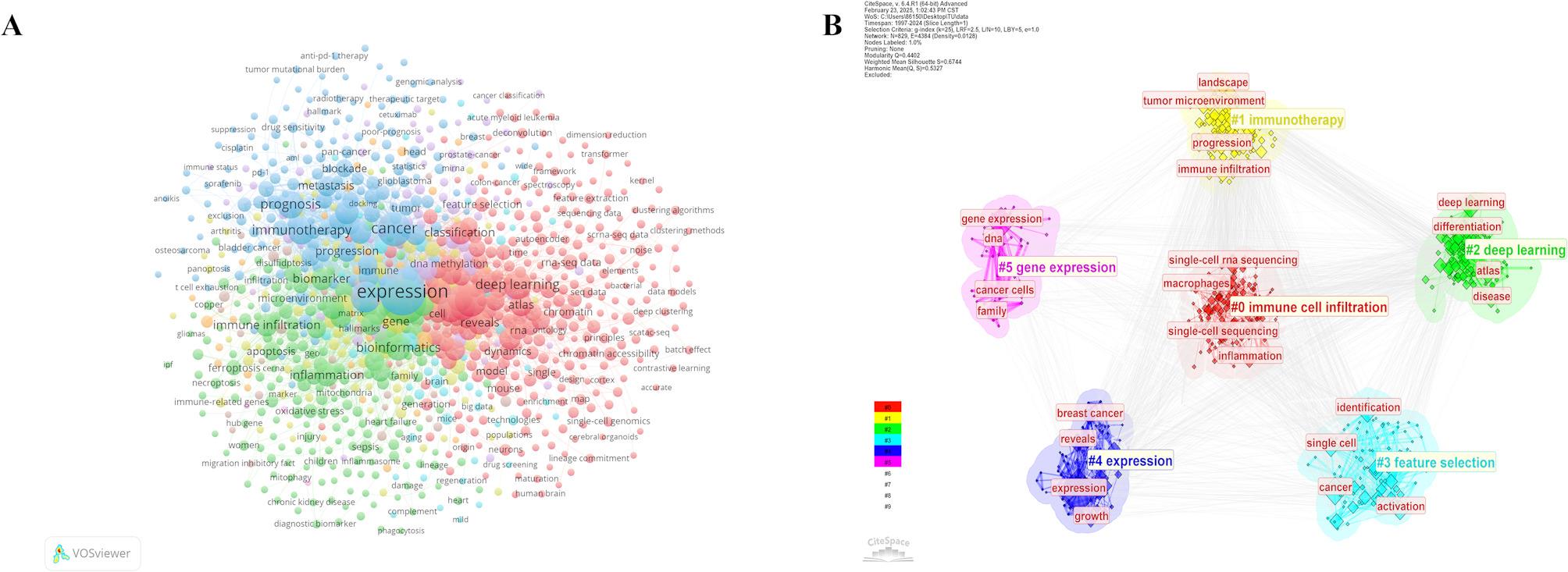
Analysis of Keyword Co-Occurrence. (**A**) Clustering and co-occurrence visualization of major keywords in Machine Learning and Single-Cell Transcriptomics research. (**B**) Domain-Specific Keyword Clustering Analysis

Keyword co-occurrence analysis serves as a key approach to identifying research hotspots, whereas burst keywords indicate emerging frontiers in ML-based SCT. Table 6 presents the top 20 most frequently occurring keywords within this domain, including their respective occurrence counts and TLS distributions. Among them, the highest-ranking keyword is “expression” (812 occurrences), followed by “machine learning” (684 occurrences) and “cancer” (337 occurrences). Additionally, terms such as “deep learning” (210 occurrences), “bioinformatics” (148 occurrences), and “single-cell” (159 occurrences) highlight the major keywords and research hotspots in this field. In VOSviewer, the keyword co-occurrence analysis for ML-based SCT is illustrated in Fig. 7A. Based on keyword proximity and co-occurrence relationships, the research landscape can be broadly divided into four clusters: Blue Cluster, primarily associated with gene expression, prognosis prediction, and immunotherapy, representing key topics such as expression, prognosis, and immunotherapy; Green Cluster, encompassing bioinformatics, immune microenvironment analysis, and the application of ML in medical data analysis, including ML, bioinformatics, and gene; Red Cluster, focused on single-cell sequencing and genomic data analysis, corresponding to classification, atlas, and single-cell genomics; Yellow Cluster, primarily related to the role of inflammation in disease, including inflammation, oxidative stress, and injury, which are linked to metabolic disorders. Notably, the green cluster connects to multiple core areas across other clusters, suggesting that these topics intersect various research disciplines.

Keywords can be categorized into research topics through clustering analysis in CiteSpace. Figure 7B displays the keyword clustering results related to ML-based SCT, identifying six primary clusters that represent major research directions: #0 Immune Cell Infiltration, which examines the role of immune cell infiltration in the tumor microenvironment, with single-cell sequencing as a primary analytical tool; #1 Immunotherapy, focusing on tumor immunotherapy, emphasizing immune infiltration and therapeutic efficacy within the tumor microenvironment; #2 Deep Learning, highlighting the role of deep learning in single-cell sequencing, cellular differentiation studies, and disease prediction; #3 Feature Selection, investigating ML techniques, particularly feature selection, in cancer research and single-cell analysis; #4 Expression, exploring gene expression patterns in breast cancer; #5 Gene Expression, examining gene regulation mechanisms and their implications in cancer progression.

**Table 6:** The top 20 keywords with the highest frequency in machine Learning-Based Single-Cell transcriptomics

Rank	Keywords	Occurrences	TLS	Rank	Keywords	Occurrences	TLS
1	expression	812	5088	11	bioinformatics	148	976
2	machine learning	684	4403	12	tumor microenvironment	131	962
3	cancer	337	2278	13	single-cell	159	945
4	gene-expression	299	1804	14	heterogeneity	147	929
5	cells	258	1665	15	scrna-seq	153	856
6	prognosis	196	1454	16	immune infiltration	119	838
7	immunotherapy	193	1439	17	reveals	134	823
8	identification	193	1292	18	inflammation	114	811
9	deep learning	210	1122	19	biomarkers	116	800
10	activation	136	993	20	gene	124	783

Note(s): Occurrences: The total number of occurrences of a keyword (or term) in a research literature collection. TLS: Total Link Strength. The total strength of connections between a keyword and all other keywords

**Fig. 8:**
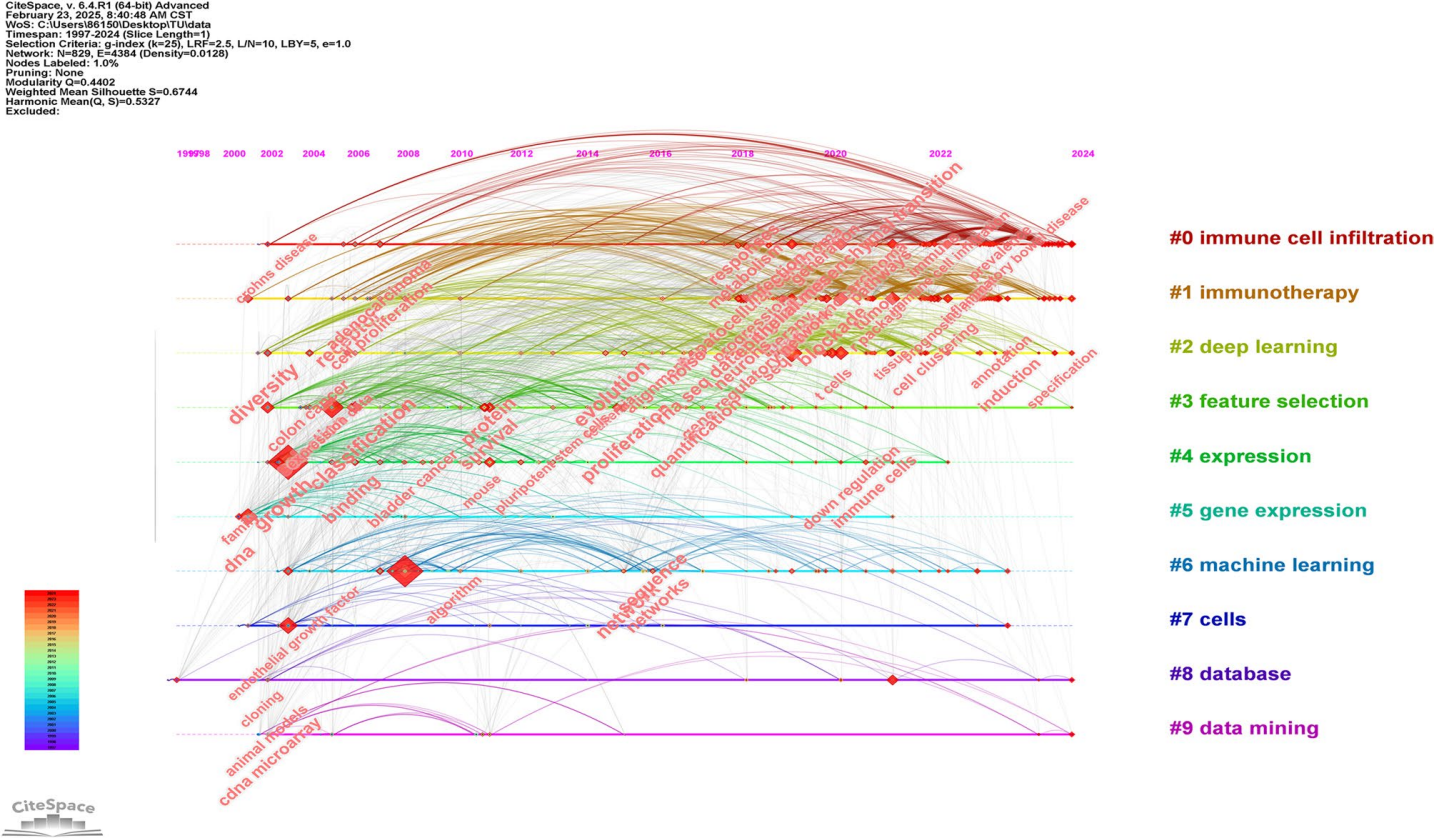
The timeline graph of keywords in CiteSpace. Each horizontal line represents a cluster. Nodes size reflects co-citation frequency, and the links between nodes indicate co-citation relationships. Nodes occurrence year is the time when they were first co-cited

CiteSpace’s timeline visualization (Fig. 8) categorizes keywords based on their first appearance, illustrating the chronological evolution of research trends. The #0 “Immune Cell Infiltration” cluster emerges as a key research area. In studies conducted from 1997 to 2010, keyword clusters were predominantly concentrated in computational fields such as #8 “Database” and #9 “Data Mining”, reflecting the focus on traditional molecular biology techniques such as cDNA microarrays. During the technology development phase from 2010 to 2016,there was a gradual shift toward ML applications, with increasing prominence of keywords such as #6 “Machine Learning” and #3 “Feature Selection”. During this phase, #5 “Gene Expression” became a central research topic. From 2018 to 2024, the most significant emerging research areas have been #0 “Immune Cell Infiltration” and #1 “Immunotherapy”. Meanwhile, #2 “Deep Learning” has expanded rapidly, integrating single-cell data for immunological and disease prediction studies. #5 “Gene Expression” and #3 “Feature Selection” continue to be essential methodologies in this domain.

Since 2010, research in single-cell transcriptomics has increasingly focused on the tumor microenvironment and immunotherapy, with immune cell infiltration and immunotherapy emerging as key themes after 2016. This shift signifies the rapid progress of the field and its growing application in clinical settings. Simultaneously, research on gene expression and cancer has transitioned from basic analysis to more integrated methodologies, particularly with the adoption of deep learning, reflecting a steady growth in the area. Since 2017, deep learning and data integration have become central to the field, especially in the analysis of high-dimensional single-cell data, showing a remarkable surge in development.

**Fig. 9:**
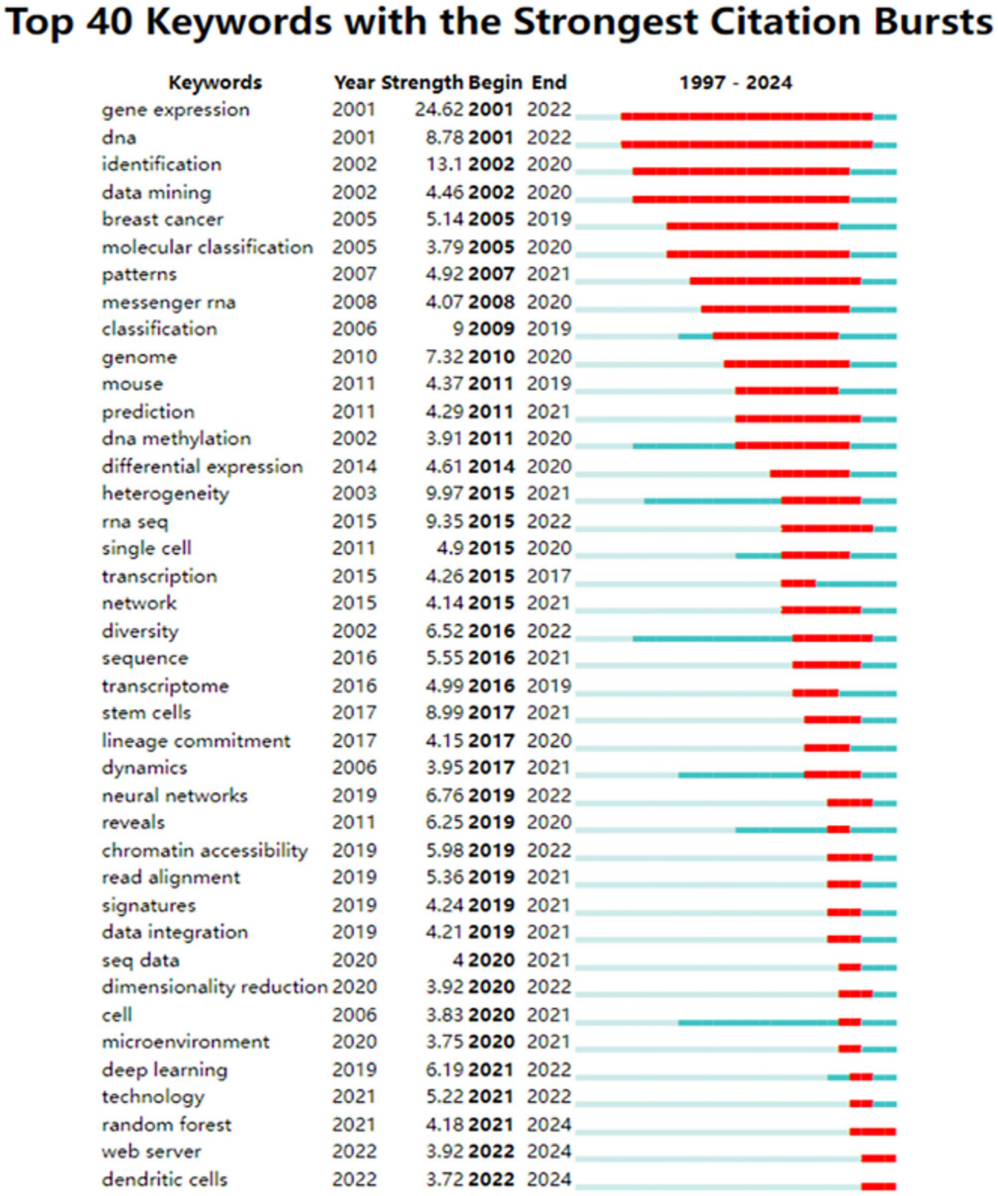
The top 40 keywords with the strongest citation bursts. The blue line indicates the time interval, and the red line indicates the period when the keyword burst occurs

The trends of these keywords can be visualized to extract the hotspots and future directions of ML-based SCT. Figure 9 highlights the top 40 most strongly cited burst keywords, revealing a timeline of rapidly increasing citations. The early studies were focused on “gene expression”, “data mining”, and “molecular classification”, which were tightly correlated to the genomic studies. Between 2010 and 2017, the field was broadened to go beyond gene expression to encompass RNA sequencing (RNA-seq), single-cell analysis, stem cells and lineage commitment, enabling important breakthroughs. Since 2018, there has been significant momentum in the application of neural networks and deep learning in bioinformatics. Simultaneously, microenvironment studies and dendritic cell related immunity research have become active fields. In addition, ML algorithms such as RF are still being rapidly developed and subsequently applied to single-cell data analysis. This analysis highlights the rapid evolution of ML in SCT, with its growing potential to impact immunology and predictive and personalized medicine.The development of foundational single-cell models, particularly exemplified by scGPT, represents a paradigm shift in cellular classification methodologies. These advanced computational frameworks enable the construction of high-fidelity cellular atlases while simultaneously facilitating the development of clinically relevant analytical pipelines, thereby providing transformative approaches for precision medicine applications.

Notably, transformer-based architectures such as CellBERT demonstrate unique capabilities in spatial transcriptomic analyses and multi-omics integration. Recent investigations (Fig. 9) reveal significantly increased adoption of multimodal fusion approaches within the research community, indicative of the remarkable progress in this technological domain.

## Discussion

### Global trends in ML-Based SCT research

This study, grounded in scientometric research spanning 1997–2024, conducts comprehensive analysis of machine learning applications in SCT to map global research trends and technological application landscapes. The innovative research paradigm achieves visualized reconstruction and systematic organization of disciplinary knowledge architecture. Particularly noteworthy is the exponential growth trajectory observed since 2018 in ML-driven SCT research outputs. The geometric expansion pattern in scholarly paper production signifies this interdisciplinary field’s transition into a new era of accelerated development.

An analysis of research output by country/region reveals that China and USA have published significantly more papers than any other countries, making them the two most influential nations in this field. This achievement is closely linked to funding institutions within these regions. Among the top ten major funding agencies, five are from China and USA. Notably, while China surpasses USA in publication volume, USA continues to dominate the field in terms of research quality, having the highest H-index and the most total citations.

Citation network analysis reveals that the United States possesses the highest TLS, suggesting a significant influence of its scientific output in this domain. This underscores the country’s strong academic performance in related research areas. In comparison, China has recognized the existing gap and has launched various measures to improve the overall quality of its academic publications [57, 58]. Remarkably, six of the world’s top ten most productive research institutions are located in China, which contributes to its dominance in publication volume within the fields of machine learning and single-cell technologies [59]. These findings emphasize the pivotal role of developing high-level research institutions in boosting national academic competitiveness.

Furthermore, among the most prolific authors in this field, Huang, Tao has made significant advances at the intersection of ML and SCT, introducing novel methods and perspectives for deciphering biological complexity at the single-cell level. By leveraging ML to enhance the analysis of scRNA-seq data [60, 61], he effectively addresses challenges such as high dimensionality, sparsity, and noise interference. His approach incorporates feature selection techniques (e.g., mRMR, Boruta) and classification algorithms (e.g., RF, deep forest, and SVM), significantly improving the accuracy of cell type identification. This strategy improves the precision of cellular heterogeneity analysis while simultaneously fostering the development of advanced computational techniques in the field of single-cell omics. By overcoming the limitations of traditional statistical methods in single-cell data analysis, Huang’s research establishes ML as a powerful tool for decoding scRNA-seq data. His contributions provide robust computational support for cell type classification, biomarker discovery [62–64], and cell fate trajectory inference, further expanding the potential of SCT. As a result, this author and their research team are likely to continue publishing highly influential studies in the future.

Among the top 10 journals analyzed, only two have an impact factor (IF) exceeding 10.0, indicating that most related studies are published in mid-tier impact factor journals. Nature Communications (IF = 14.7) and Genome Biology (IF = 10.1) stand out as the most influential journals in this field, typically favoring research with broad biological significance or major technological innovations. These journals tend to prioritize studies that introduce novel single-cell data analysis methods or uncover key mechanisms underlying specific diseases.

Machine learning technologies provide critical computational support for SCT data analysis. To address the challenges of high-dimensionality and noise in scRNA-seq data, deep learning and ensemble learning methods significantly improve the accuracy of cell classification and annotation, overcoming the limitations of traditional dimensionality reduction techniques [65, 66]. In cell trajectory inference, the integration of reinforcement learning with RNA velocity models optimizes dynamic process analysis, supporting mechanistic exploration in fields such as cancer immunotherapy [67]. Furthermore, multi-omics integration studies enhance compatibility across data types and platforms through generative adversarial networks (GANs) and Transformer models, laying the foundation for cross-species research and clinical translation [68]. Large ML models, especially Transformers, demand heavy hardware and memory, causing long training times and high costs. Real-time inference is slow, and memory-saving can hurt accuracy. Training is unstable and energy use is high, raising environmental concerns. Balancing these issues is challenging [69, 70].

However, transparency in machine learning–based research still needs improvement. In prognostic modeling, for instance, emerging AI/ML algorithms offer promising support for clinical decision-making in intensive care units (ICUs), but their inner workings are often opaque [72]. Clinicians may lack a clear understanding of how these models generate predictions, which can hinder trust and practical adoption. Moreover, the use of ML tools in clinical trials poses new regulatory challenges, with transparency around data and algorithms being a central concern [73]. In some studies, limited disclosure of algorithmic details, data sources, and preprocessing methods increases regulatory complexity and undermines the credibility and generalizability of the findings.

Reproducibility remains a significant challenge in ML-SCT research. Variability in experimental protocols can lead to inconsistent results. For instance, in studies of microbial diversity, differences in cell lysis techniques, PCR primers, and computational analysis methods can cause substantial variation in microbial community profiling [74]. One study examined chicken manure fermentation samples using four distinct lysis methods, three commonly used primer sets, and multiple computational pipelines. The resulting microbial compositions varied widely depending on the approach, highlighting serious reproducibility concerns.

Similar issues arise in data analysis. In somatic point mutation detection, discrepancies across sequencing platforms, strategies, and variant calling tools can lead to inconsistent findings [75]. A study using Coriell cell lines to mimic clinical biopsy samples evaluated two targeted sequencing panels and multiple variant callers. It found a high number of false positives—many of them non-overlapping—across different variant calling methods. Although certain approaches helped reduce this inconsistency, false positive rates remained substantial. These findings underscore the need for more rigorous standardization across all stages of ML-SCT research—from experimental design to computational analysis—to ensure reproducibility and reliability of results.

Currently, machine learning is driving the transition of single-cell studies from methodological development to clinical applications, though challenges such as computational complexity and standardized evaluation frameworks require further resolution [76, 71].

### Hot topics and emerging trends in ML and SCT research

In scientific research, one of the key evaluation metrics is the citation frequency, meaning the total number of times a research paper is cited by others during a specific time range. The active development of new ML methodologies, which draws from data to render better inferences on biological systems, may keep SCT studies relevant long after they are no longer at the cutting edge of biological discoveries, meaning that high citation frequency may not be indicative of an obsolete field but rather one that researchers have an enduring interest and attention in, particularly as new ML methods are developed and asked to render better conclusions on heterogenous biological systems. The analysis shows that nearly nine out of ten key references achieved their highest citation frequencies during 2015 to 2024, marking a decade of accelerated expansion in this research domain.

Recent research hotspots related to “dendritic cells,” “web servers,” and “random forest” in this study by keyword co-occurrence analysis and timeline clustering. These latter subjects illustrate the increasing prevalence of ML to manage individual cell datasets, such as immune cell types, computational visualization tools, and predictive modeling [77]. As these technologies develop, they play an evermore crucial role in the progression of SCT.

#### Random forest

The RF method is an ensemble ML method that relies on creating decision trees and is commonly used for classification, feature selection, and predictive analysis in Bioinformatics [78]. Its main advantages are in its capacity to work with high-dimensional data to suppress/mitigate overfitting and provide good generalization [79]. The application of RF in SCT in recent years has made tremendous progress, especially in cell type identification, feature gene selection, and disease state prediction [80, 81].

For cell type classification, Random Forest can recognize different gene expression patterns, so different cell subpopulations could be identified by sequencing. In fact, it has been applied to classify single-cell data in general in Tumor Microenvironment research, by distinguishing immune cell subtypes and predicting their functional states [80]. This technology is essential for analyzing the heterogeneity of immune cells in tumors and theoretically supports the development of targeted immunotherapies.

Due to the feature importance evaluation mechanism of RF, RF is skillful in obtaining the important gene associated with disease progression, making researchers identify the candidate genes easier through feature gene selection [79]. The approach is widely applied in autoimmune disease research through the identification of core genes that drive the onset and development of disease, thereby providing novel biomarkers for the formulation of personalized treatment strategies [77]. Moreover, RF has been broadly applied in single-cell immune analysis to aid identifying target key genes that affect T cells, DCs, and macrophages [82].

RF has also been a vital instrument in predictive medicine in disease, making it essential in precision medicine. By integrating single-cell data, the researchers can construct predictive models to approximate a patient’s immune status, disease state, or response to treatment [83]. As such, RF models have been used to predict the responses of patients with cancer to immune checkpoint inhibitors (e.g., PD-1/PD-L1 antibodies), thus providing useful information for clinical decision-making. RF also helps to predict transitions in cell fate [84], and so provides a better understanding of cell differentiation.

For GRN reconstruction, RF can reconstruct networks of interacting genes that enable the researchers to determine the cell fate [85]. It has been used to discover major regulating genes of dendritic cells in tumor microenvironment studies, as well as to identify candidate immune regulatory pathways.

However, with the introduction of various deep learning methods, the use of hybrid models which combine RF with neural networks/tools, has become a trend in single-cell bioinformatics [86]. They have been used in cancer immunology, to classify immune cells more accurately, and at a lower cost, than using such models separately. A combination of computer-aided and conventional hand-derived measures can improve the accuracy of immune cell subpopulation classification, while still improving disease prediction and treatment response evaluations [87]. Moreover, as ML methods keep progressing forward, such synergy of RF with deep learning should power further advances in SCT and immunology research.

#### Web servers

Many bioinformatics data sharing and online analysis services rely heavily on web servers, including, but not limited to, visualization, interactive exploration and automated processing of single-cell data [88]. Integrating with their respective platforms, however, has allowed for single cell transcriptomic data to be more accessible and usable than ever before [89]. This approach supports data handling for big data sets, encompassing various gene expression databases like Gene Expression Omnibus, The Cancer Genome Atlas, and Human Protein Atlas (HPA) without the need for storage, and offering advanced online query tool and computational tools to researchers [90]. They can allow comparing and analysis of gene expression on different cell types, as in HPA [91]. Furthermore, visualization software such as the Xena Browser aids the exploration of single cell data, assisting researchers in the discovery of previously hidden features from datasets.

In addition, an increasing number of Web servers use ML algorithms due to technology advancement, which allows them to automatically perform analyses like cell classification, gene feature extraction, and disease prediction [92]. Cell Marker, for instance, is a comprehensive database dedicated to the deposition and query of single-cell markers from a wide range of tissues, allowing for deep interrogation of large single-cell datasets for the retrieval of relevant cell populations [93]. while a ML tool known as single-cell regulatory network inference and clustering, built on the RF algorithm, enables in-depth analysis of single-cell GRN, providing remote computation support using web-based platforms [94].

Since single-cell data is high-dimensional, Web servers also offer visualization tools (such as t-SNE and UMAP) for data exploration so that users do not need extensive programming experience [95]. Web-based platforms such as Seurat and Scamp which can perform online SCT analysis and interactively visualize the result data have been developed to assist the user [96, 97].

In future, we also see deep learning and cloud computing further integrated on a web server for automation of data analysis and scalability [98]. Such developments will offer solid computational bases for personalized medicine and precision therapeutics through Web-based platforms and will continuously position them at the forefront of biomedical research and data-driven discoveries.

#### Dendritic cells

Dendritic cells (DCs) are a major cell type of the adaptive immune system responsible for antigen uptake and processing as well as presentation to T cells [99]. SCT development has allowed researchers to obtain more detailed information on the functional heterogeneity of DCs, which has also accelerates the progress of immunological studies [100].

Regarding the analysis of DC subpopulations, the scRNA-seq technology is useful for specific identification of various DC subsets, like the cDC1, cDC2, and pDCs [101]. The cDC1 subpopulation is mainly responsible for antigen presentation to CD8 + T cells and mediates anti-tumor immunity in this context [102]. Compared with cDC2 that predominantly activates CD4 + T cells to induce immune response and vaccine-mediated immunity. On the other hand, pDCs play a central role in antiviral immunity by secreting the most potent type I interferons (IFN-α/β), which potentiate the antiviral response at the level of the host [103]. DCs in the TME are well characterized and exhibit suppressed function, displaying a diminished capacity to present antigen and impairing immune responses transfer studies in vivo [104]. Comprehension of these persistent shifts in the DC population compartment in TME promotes tailored approaches to tumor immunotherapy.

ML, in particular RF, has been extensively used in DC-related studies [105]. Combining with scRNA-seq data, the RF model can help predict the distribution of DC subsets in different tumor types, thereby suggesting theoretical basis for personalized cancer immunotherapy [106]. Furthermore, dendritic cell vaccines (DCVax) as a DC-based immunotherapy paradigm have emerged as a potent candidate in cancer therapy [107]. SCT data integrated with ML algorithms could provide a basis for the further refinement of personalized DC-based vaccines. This metallic performance is compatible with VISTA usage at immune checkpoints as well as antigen-presenting capabilities of dendritic cells (DC) in competition to solve optimal vaccine formulations for distinct cancer types and increase both vaccine efficiency and therapeutic outcomes through Web servers interfaced with RF algorithms to globally dissect SCT data for the most appropriate DC subsets selection for the use of a VISTA-based vaccine development [108].

As ML advances, the convergence of single-cell sequencing and computational modeling will increasingly refine precision immunotherapy, enabling more targeted dendritic cell-based therapeutic strategies.

### Limitations of single-cell transcriptomics and machine learning

SCT has significantly advanced biological research by providing unparalleled resolution and insights into cellular diversity. However, the application of machine learning techniques within this domain is not without its challenges. One of the primary concerns is the use of black-box models, which often lack interpretability. This lack of transparency restricts their use in biomedical applications, where understanding the underlying mechanisms driving predictions is critical. While machine learning models, particularly deep learning, are highly effective at processing large datasets and generating accurate predictions, their opaque nature makes it difficult to decipher the rationale behind these predictions, posing a challenge in fields like clinical decision-making and diagnostics that demand clarity and trust in the model’s outputs.

Moreover, machine learning methods often struggle with issues such as poor generalization and an over-reliance on feature engineering, which complicates their application to complex biological datasets, especially those with limited sample sizes. In single-cell transcriptomics, the heterogeneity of individual cells, combined with the inherent noise in sequencing data, adds another layer of complexity [109]. These factors often require the integration of multiple technologies to enhance data quality and reduce bias, which further complicates model development [110].

Additionally, the integration of SCT with other omics layers remains underexplored. While significant advances have been made in transcriptomic analysis, incorporating data from proteomics and metabolomics is still relatively limited. This lack of multi-omics integration impedes a more holistic understanding of cellular processes and disease mechanisms.

### Limitation

Although it offers notable contributions, this study has certain limitations. Firstly, the exclusive reliance on WoSCC database, while demonstrating technical compatibility and disciplinary representativeness, risks systematic omissions of literature from heterogeneous repositories like PubMed and Scopus. Secondly, terminological inconsistencies manifested through synonym variations and acronym ambiguities may introduce structural biases in keyword co-occurrence networks. Thirdly, the limitation to English-language publications introduces both linguistic and geographical biases, which may result in the exclusion of important findings from non-English-speaking regions.Moreover, existing studies may be subject to technical and algorithmic biases.The exclusion of literature in all languages may result in some bias.

Additionally, we suggest that future reviews integrate full-text NLP analysis to capture detailed methodological insights beyond what is provided in the metadata. This approach would enable a more thorough evaluation of research methodologies and offer a deeper understanding of the underlying processes across studies.

## Conclusions

The integration of ML with SCT has greatly enhanced the study of cellular heterogeneity, regulatory networks, and disease mechanisms. Over the past few years, advancements in these domains have been driven by cross-disciplinary collaboration between computational and biomedical experts, particularly facilitated by collaborations between China and USA. Current priorities focus on three key directions: deep learning approaches for high-dimensional transcriptomic data analysis, spatial transcriptomics for tissue mapping, and characterization of the immune microenvironment. However, current challenges hinder the wider applicability of these technologies. The heterogeneities challenge the comparability between studies, the fusion of heterogeneous data remains a technical challenge, and the limited interpretability of models hampers their clinical adoption. The implications are that advances in generalizable models, computational scalability, and the development of tools for model interpretability are crucial for addressing these challenges. In the future, research efforts should prioritize the refinement of deep learning models, the enhancement of their cross-domain generalization capabilities, and the expansion of ML use cases in single-cell biological studies. Continued integration across disciplines will be key to unlocking the full potential of machine learning in SCT and accelerating progress in precision medicine.

## Supplementary Information

Below is the link to the electronic supplementary material.

Supplementary Material 1

Supplementary Material 2

Supplementary Material 3

## Data Availability

No datasets were generated or analysed during the current study.

## Abbreviations

ML: Machine learning
scRNA-seq: Single-cell RNA sequencing
WOSCC: Web of Science Core Collection
SCT: Single-Cell Transcriptomics
TLS: Total Link Strength
TS: Topic Search
MCP: Multinational Cooperation Pap

## References

[1] Stuart T et al. *Comprehensive integration of single-cell data.* 2019. 177(7): pp. 1888–1902. e21. 10.1016/j.cell.2019.05.031 PMC6687398 31178118

[2] Chen X et al. Top-100 highest-cited original articles in inflammatory bowel disease: A bibliometric analysis. 2019. 98(20): p. e15718. 10.1097/MD.0000000000015718 PMC6531102 31096525

[3] Hwang B et al. *Single-cell RNA sequencing technologies and bioinformatics pipelines.* 2018. 50(8): pp. 1–14. 10.1038/s12276-018-0071-8 PMC6082860 30089861

[4] Wani SA, Khan SA. and S.J.A.o.C.M.i.E. Quadri, *Application of deep learning for single cell multi-omics: a state-of-the-art review.* 2025: pp. 1–43.

[5] Jin S et al. Inference and analysis of cell-cell communication using cellchat. 2021. 12(1): p. 1088. 10.1038/s41467-021-21246-9 PMC7889871 33597522

[6] Stetson L et al. Single cell RNA sequencing of AML initiating cells reveals RNA-based evolution during disease progression. 2021. 35(10): pp. 2799–812. 10.1038/s41375-021-01338-7 PMC8807029 34244611

[7] Wolf FA, Angerer P. and F.J.J.G.b. Theis, *SCANPY: large-scale single-cell gene expression data analysis.* 2018. 19: pp. 1–5. 10.1186/s13059-017-1382-0 PMC5802054 29409532

[8] Luecken MD, F.J.J. .M.s.b. Theis, *Current best practices in single-cell RNA‐seq analysis: a tutorial*. 2019. 15(6): p. e8746. 10.15252/msb.20188746 PMC6582955 31217225

[9] Petegrosso R, Li Z. and R.J.B.i.b. Kuang, *Machine learning and statistical methods for clustering single-cell RNA-sequencing data*. 2020. 21(4): pp. 1209–23. 10.1093/bib/bbz063 31243426

[10] Sha Y et al. *Reconstructing growth and dynamic trajectories from single-cell transcriptomics data.* 2024. 6(1): pp. 25–39. 10.1038/s42256-023-00763-w PMC10805654 38274364

[11] Patel M et al. *Advances in machine learning, statistical methods, and ai for single-cell rna annotation using raw count matrices in scrna-seq data.* 2024.

[12] Brendel M et al. Application of deep learning on single-cell RNA sequencing data analysis: a review. 2022. 20(5): pp. 814–35. 10.1016/j.gpb.2022.11.011 PMC10025684 36528240

[13] Wilk AJ et al. A single-cell atlas of the peripheral immune response in patients with severe COVID-19. 2020. 26(7): pp. 1070–6. 10.1038/s41591-020-0944-y PMC7382903 32514174

[14] Sade-Feldman M et al. Defining T cell States associated with response to checkpoint immunotherapy in melanoma. 2018. 175(4): pp. 998–1013. e20. 10.1016/j.cell.2018.10.038 PMC6641984 30388456

[15] Flores M et al. Deep learning tackles single-cell analysis—a survey of deep learning for scRNA-seq analysis. 2022. 23(1): p. bbab531. 10.1093/bib/bbab531 PMC8769926 34929734

[16] Tejada-Lapuerta A et al. *Causal machine learning for single-cell genomics.* 2023. 10.1038/s41588-025-02124-2 40164735

[17] Liu J et al. *Machine intelligence in single-cell data analysis: advances and new challenges.* 2021. 12: p. 655536. 10.3389/fgene.2021.655536 PMC8203333 34135939

[18] Chen L et al. *Bibliometric and visual analysis of single-cell sequencing from 2010 to 2022.* 2024. 14: p. 1285599. 10.3389/fgene.2023.1285599 PMC10808606 38274109

[19] Wang J et al. Bibliometric and visual analysis of single-cell multiomics in neurodegenerative disease arrest studies. 2024. 15: p. 1450663. 10.3389/fneur.2024.1450663 PMC11493674 39440247

[20] Ma T et al. *Artificial intelligence and machine (Deep) learning in otorhinolaryngology: A bibliometric analysis based on VOSviewer and citeSpace.* 2023: p. 01455613231185074. 10.1177/01455613231185074 37515527

[21] Luo Z, Lv J, Zou KJFiM. Bibliometric Anal Artif Intell Res Crit Illness: Quant Approach Visualization Study. 2025;12:1553970. 10.3389/fmed.2025.1553970 PMC11914116 40103796

[22] Lundberg L et al. *Bibliometric Mining of Research Trends in Machine Learning.* 2024. 5(1): pp. 208–236.

[23] Luo C et al. *The research hotspots and theme trends of artificial intelligence in nurse education: A bibliometric analysis from 1994 to 2023.* 2024: p. 106321. 10.1016/j.nedt.2024.106321 39084073

[24] Malele V. Evaluations Large Lang Models Bibliometric Anal 2024. 13(1).

[25] Kargozar S et al. *Bioactive glasses: sprouting angiogenesis in tissue engineering.* 2018. 36(4): pp. 430–444. 10.1016/j.tibtech.2017.12.003 29397989

[26] Meho LIJPW. Rise Rise Cit Anal. 2007;20(1):32.

[27] Falagas ME et al. *Comparison of PubMed, Scopus, web of science, and Google scholar: strengths and weaknesses.* 2008. 22(2): pp. 338–342. 10.1096/fj.07-9492LSF 17884971

[28] Perazzo MF et al. *The top 100 most-cited papers in Paediatric Dentistry journals: A bibliometric analysis.* 2019. 29(6): pp. 692–711. 10.1111/ipd.12563 31325392

[29] Dong R et al. *Publication trends for Alzheimer’s disease worldwide and in China: a 30-year bibliometric analysis.* 2019. 13: p. 259. 10.3389/fnhum.2019.00259 PMC6696880 31447661

[30] Chen CJ. J.o.t.A.S.f.i.S. And technology, *CiteSpace II: detecting And visualizing emerging trends And transient patterns in scientific literature*. 2006. 57(3): pp. 359–77.

[31] Börner K, et al. Visualizing Knowl Domains. 2003;37(1):179–255.

[32] Liu H-C, Sung W-P, Yao W. Information technology and computer application engineering. CRC; 2014.

[33] Van Eck NJ. and L.J.a.p.a. Waltman, *Text mining and visualization using VOSviewer.* 2011.

[34] Van Eck N, Waltman LJs. *Software survey: VOSviewer, a computer program for bibliometric mapping.* 2010. 84(2): pp. 523–538. 10.1007/s11192-009-0146-3 PMC2883932 20585380

[35] Van Eck NJ, Waltman LJUL. *VOSviewer Manual: Version 1.6. 5.* 2016.

[36] Šubelj L, Van Eck NJ, Waltman LJPo. Clustering Sci Publications Based Cit Relations: Syst Comparison Different Methods. 2016;11(4):e0154404. 10.1371/journal.pone.0154404 PMC4849655 27124610

[37] Page MJ et al. *PRISMA 2020 explanation and elaboration: updated guidance and exemplars for reporting systematic reviews.* 2021. 372. 10.1136/bmj.n160 PMC8005925 33781993

[38] Chen C, Song MJPo. Visualizing Field Research: Methodol Syst Scientometr Reviews. 2019;14(10):e0223994. 10.1371/journal.pone.0223994 PMC6822756 31671124

[39] Van Raan. A.J.S.h.o.s. and t. indicators, *Measuring science: Basic principles and application of advanced bibliometrics.* 2019: pp. 237–280.

[40] Leydesdorff L. J.a.p.a., *Eugene Garfield and algorithmic historiography: Co-words, co-authors, and journal names.* 2010.

[41] Aria M. and C.J.J.o.i. Cuccurullo, *bibliometrix: an R-tool for comprehensive science mapping analysis*. 2017. 11(4): pp. 959–75.

[42] Shen Z et al. *The global research of artificial intelligence on prostate cancer: a 22-year bibliometric analysis.* 2022. 12: p. 843735. 10.3389/fonc.2022.843735 PMC8921533 35299747

[43] Hänzelmann S, Castelo R, Guinney JJBb. *GSVA: gene set variation analysis for microarray and RNA-seq data.* 2013. 14: pp. 1–15. 10.1186/1471-2105-14-7 PMC3618321 23323831

[44] Korsunsky I et al. *Fast, sensitive and accurate integration of single-cell data with Harmony.* 2019. 16(12): pp. 1289–1296. 10.1038/s41592-019-0619-0 PMC6884693 31740819

[45] Langfelder P, Horvath SJBb. WGCNA: R Package Weighted Correlation Netw Anal. 2008;9(1):559. 10.1186/1471-2105-9-559 PMC2631488 19114008

[46] Hänzelmann S, Castelo R. and J.J.B.b. Guinney, *GSVA: gene set variation analysis for microarray and RNA-seq data*. 2013. 14(1): p. 7. 10.1186/1471-2105-14-7 PMC3618321 23323831

[47] Wolf FA, Angerer P. and F.J.J.G.b. Theis, *SCANPY: large-scale single-cell gene expression data analysis.* 2018. 19(1): p. 15. 10.1186/s13059-017-1382-0 PMC5802054 29409532

[48] Yu G et al. ClusterProfiler: an R package for comparing biological themes among gene clusters. 2012. 16(5): pp. 284–7. 10.1089/omi.2011.0118 PMC3339379 22455463

[49] Ritchie ME et al. *limma powers differential expression analyses for RNA-sequencing and microarray studies.* 2015. 43(7): pp. e47-e47. 10.1093/nar/gkv007 PMC4402510 25605792

[50] Zhang Y et al. Single-cell analyses reveal key immune cell subsets associated with response to PD-L1 Blockade in triple-negative breast cancer. 2021. 39(12): pp. 1578–93. e8. 10.1016/j.ccell.2021.09.010 34653365

[51] Zhang S, et al. Single Cell Transcriptomic Analyses Implicate Immunosuppressive Tumor Microenvironment Pancreat Cancer Liver Metastasis. 2023;14(1):5123. 10.1038/s41467-023-40727-7 PMC10447466 37612267

[52] Zhang L, et al. Clin Translational Values Spat Transcriptomics. 2022;7(1):111. 10.1038/s41392-022-00960-w PMC8972902 35365599

[53] Abdelaal T et al. A comparison of automatic cell identification methods for single-cell RNA sequencing data. 2019. 20(1): p. 194. 10.1186/s13059-019-1795-z PMC6734286 31500660

[54] Nair SS et al. The tumor microenvironment and immunotherapy in prostate and bladder cancer. 2020. 47(4): pp. e17–54. 10.1016/j.ucl.2020.10.005 33446323

[55] Wu Z et al. Graph deep learning for the characterization of tumour microenvironments from Spatial protein profiles in tissue specimens. 2022. 6(12): pp. 1435–48. 10.1038/s41551-022-00951-w 36357512

[56] Jiang P et al. Signatures of T cell dysfunction and exclusion predict cancer immunotherapy response. 2018. 24(10): pp. 1550–8. 10.1038/s41591-018-0136-1 PMC6487502 30127393

[57] Verwoerd L et al. *Negotiating space for knowledge co-production.* 2023. 50(1): pp. 59–71.

[58] Wang Q et al. *Characterization of global research trends and prospects on single-cell sequencing technology: bibliometric analysis.* 2021. 23(8): p. e25789. 10.2196/25789 PMC8386406 34014832

[59] Ayad LA, Charalampopoulos P, Pissis SPJB. SMART: SuperMaximal Approximate Repeats Tool. 2020;36(8):2589–91. 10.1093/bioinformatics/btz953 31873724

[60] Huang F, et al. Identification of human cell cycle phase markers based on Single-Cell RNA‐Seq. Data Using Mach Learn Methods. 2022;2022(1):2516653. 10.1155/2022/2516653 PMC9393965 36004205

[61] Li Z et al. Identifying in vitro cultured human hepatocytes markers with machine learning methods based on single-cell RNA-Seq data. 2022. 10: p. 916309. 10.3389/fbioe.2022.916309 PMC9189284 35706505

[62] Lu J et al. Identification of COVID-19 severity biomarkers based on feature selection on single-cell RNA-Seq data of CD8 + T cells. 2022. 13: p. 1053772. 10.3389/fgene.2022.1053772 PMC9682094 36437952

[63] Moreno P et al. User-friendly, scalable tools and workflows for single-cell RNA-seq analysis. 2021. 18(4): pp. 327–8. 10.1038/s41592-021-01102-w PMC8299072 33782609

[64] Lopez R et al. Deep generative modeling for single-cell transcriptomics. 2018. 15(12): pp. 1053–8. 10.1038/s41592-018-0229-2 PMC6289068 30504886

[65] Hao Y et al. Integrated analysis of multimodal single-cell data. 2021. 184(13): pp. 3573–87. e29. 10.1016/j.cell.2021.04.048 PMC8238499 34062119

[66] Li X et al. Deep learning enables accurate clustering with batch effect removal in single-cell RNA-seq analysis. 2020. 11(1): p. 2338. 10.1038/s41467-020-15851-3 PMC7214470 32393754

[67] Bergen V et al. Generalizing RNA velocity to transient cell States through dynamical modeling. 2020. 38(12): pp. 1408–14. 10.1038/s41587-020-0591-3 32747759

[68] Ahmed KT, et al. Multi-omics Data Integr Generative Adversarial Netw. 2022;38(1):179–86. 10.1093/bioinformatics/btab608 PMC10060730 34415323

[69] Kaplan J et al. *Scaling laws for neural language models.* 2020.

[70] Vaswani A et al. Atten Is all You Need 2017. 30.

[71] Soll RF, Ovelman C. J.E.h.d. McGuire. Future Cochrane Neonatal. 2020;150:105191. 10.1016/j.earlhumdev.2020.105191 33036834

[72] Weissman GE. and V.X.J.C.o.i.c.c. Liu, *Algorithmic prognostication in critical care: A promising but unproven technology for supporting difficult decisions.* 2021. 27(5): pp. 500–505. 10.1097/MCC.0000000000000855 PMC8416806 34267077

[73] Massella M et al. Regulatory considerations on the use of machine learning based tools in clinical trials. 2022. 12(6): pp. 1085–96. 10.1007/s12553-022-00708-0 PMC9638313 36373014

[74] Krakat N, et al. Methodological Flaws Introduce Strong Bias into Mol Anal Microb Populations. 2017;122(2):364–77. 10.1111/jam.13365 27914209

[75] Karimnezhad A et al. *Accuracy and reproducibility of somatic point mutation calling in clinical-type targeted sequencing data.* 2020. 13(1): p. 156. 10.1186/s12920-020-00803-z PMC7560075 33059707

[76] Argelaguet R et al. Computational principles and challenges in single-cell data integration. 2021. 39(10): pp. 1202–15. 10.1038/s41587-021-00895-7 33941931

[77] Zhou X et al. *DeepIMAGER: Deeply Analyzing Gene Regulatory Networks from scRNA-seq Data.* 2024. 14(7): p. 766. 10.3390/biom14070766 PMC11274664 39062480

[78] Breiman LJMl. Random Forests. 2001;45:5–32.

[79] Zhao Y et al. *Rfcell: A gene selection approach for scrna-seq clustering based on permutation and random forest.* 2021. 12: p. 665843. 10.3389/fgene.2021.665843 PMC8354212 34386033

[80] Lu M et al. *LR hunting: A random forest based cell–cell interaction discovery method for single-cell gene expression data.* 2021. 12: p. 708835. 10.3389/fgene.2021.708835 PMC8420858 34497635

[81] Liu A et al. Discovery of cell type classification marker genes from single cell RNA sequencing data using NS-Forest. 2024. 10.1186/s44330-024-00015-2 PMC12396544 40893796

[82] Aybey B, et al. Immune Cell Type Signature Discovery Random for Classif Anal Single Cell Gene Expression Datasets. 2023;14:1194745. 10.3389/fimmu.2023.1194745 PMC10441575 37609075

[83] Sun B et al. Single-cell RNA sequencing in cancer research: discovering novel biomarkers and therapeutic targets for immune checkpoint Blockade. 2023. 23(1): p. 313. 10.1186/s12935-023-03158-4 PMC10704754 38066642

[84] Malidarreh PB et al. *Predicting Future States with Spatial Point Processes in Single Molecule Resolution Spatial Transcriptomics.* 2024.

[85] Akers K, T.J.C.O.i.S B, Murali. Gene Regul Netw Inference single-cell Biology. 2021;26:87–97.

[86] Erfanian N, et al. Deep Learn Appl single-cell Genomics Transcriptomics Data Anal. 2023;165:115077. 10.1016/j.biopha.2023.115077 37393865

[87] Tietscher S et al. *A comprehensive single-cell map of T cell exhaustion-associated immune environments in human breast cancer.* 2023. 14(1): p. 98. 10.1038/s41467-022-35238-w PMC9822999 36609566

[88] Skinner OS. And n.l.j.n.b. Kelleher. Illuminating Dark Matter Shotgun Proteom. 2015;33(7):717–8. 10.1038/nbt.3287 26154010

[89] Yu D et al. TIR domains of plant immune receptors are 2′, 3′-cAMP/cGMP synthetases mediating cell death. 2022. 185(13): pp. 2370–86. e18. 10.1016/j.cell.2022.04.032 35597242

[90] Li Q et al. The impact of mutations in SARS-CoV-2 Spike on viral infectivity and antigenicity. 2020. 182(5): pp. 1284–94. e9. 10.1016/j.cell.2020.07.012 PMC7366990 32730807

[91] Yu H et al. A route to de Novo domestication of wild allotetraploid rice. 2021. 184(5): pp. 1156–70. e14. 10.1016/j.cell.2021.01.013 33539781

[92] Hu C et al. *CellMarker 2.0: an updated database of manually curated cell markers in human/mouse and web tools based on scRNA-seq data.* 2023. 51(D1): pp. D870-D876. 10.1093/nar/gkac947 PMC9825416 36300619

[93] Aibar S, et al. SCENIC: single-cell Regul Netw Inference Clustering. 2017;14(11):1083–6. 10.1038/nmeth.4463 PMC5937676 28991892

[94] Bravo González-Blas C et al. CisTopic: cis-regulatory topic modeling on single-cell ATAC-seq data. 2019. 16(5): pp. 397–400. 10.1038/s41592-019-0367-1 PMC6517279 30962623

[95] Krassowski M et al. *State of the field in multi-omics research: from computational needs to data mining and sharing.* 2020. 11: p. 610798. 10.3389/fgene.2020.610798 PMC7758509 33362867

[96] Rich JM et al. *The impact of package selection and versioning on single-cell RNA-seq analysis.* 2024. 10.1016/j.cels.2026.101560 41903535

[97] Molho D, et al. Deep Learn single-cell Anal. 2024;15(3):1–62.

[98] Sachdeva S et al. *Unraveling the role of cloud computing in health care system and biomedical sciences.* 2024. 10.1016/j.heliyon.2024.e29044 PMC11004887 38601602

[99] Barut GT et al. Single-cell transcriptomics reveals striking heterogeneity and functional organization of dendritic and monocytic cells in the bovine mesenteric lymph node. 2023. 13: p. 1099357. 10.3389/fimmu.2022.1099357 PMC9853064 36685557

[100] Gao Y et al. Single-cell analysis reveals the heterogeneity of monocyte-derived and peripheral type-2 conventional dendritic cells. 2021. 207(3): pp. 837–48. 10.4049/jimmunol.2100094 34282004

[101] Gerhard GM, et al. Tumor-infiltrating Dendritic Cell States Are Conserved Solid Hum Cancers. 2020;218(1):e20200264. 10.1084/jem.20200264 PMC7754678 33601412

[102] Maier B et al. A conserved dendritic-cell regulatory program limits antitumour immunity. 2020. 580(7802): pp. 257–62. 10.1038/s41586-020-2134-y PMC7787191 32269339

[103] Chen C, et al. Tumor microenvironment-mediated Immune Evasion Hepatocellular Carcinoma. 2023;14:1133308. 10.3389/fimmu.2023.1133308 PMC9950271 36845131

[104] Xiao Z, et al. Impaired Function Dendritic Cells Within Tumor Microenvironment. 2023;14:1213629. 10.3389/fimmu.2023.1213629 PMC10333501 37441069

[105] Aevermann B et al. A machine learning method for the discovery of minimum marker gene combinations for cell type identification from single-cell RNA sequencing. 2021. 31(10): pp. 1767–80. 10.1101/gr.275569.121 PMC8494219 34088715

[106] Burns JJNRDD. *Upcoming catalysts in Q2 2015.* 2015. 14(4): pp. 228–229. 10.1038/nrd4598 25764988

[107] Ma Z et al. Single-cell sequencing analysis and multiple machine-learning models revealed the cellular crosstalk of dendritic cells and identified FABP5 and KLRB1 as novel biomarkers for psoriasis. 2024. 15: p. 1374763. 10.3389/fimmu.2024.1374763 PMC11002082 38596682

[108] Ye Z et al. Integrating bulk and Single-Cell RNA-Seq data to identify prognostic features related to activated dendritic cells in Clear-Cell Renal-Cell carcinoma. 2024. 25(17): p. 9235. 10.3390/ijms25179235 PMC11395106 39273185

[109] Dulin JN, et al. Transcriptomic Approaches Neural Repair. 2015;35(41):13860–7. 10.1523/JNEUROSCI.2599-15.2015 PMC4604224 26468186

[110] Avrahami D, et al. Beta Cell Heterogeneity: Evol Concept. 2017;60(8):1363–9. 10.1007/s00125-017-4326-z PMC5554543 28597073

