# Supplementary material for: Global trends in machine learning applications for single-cell transcriptomics research
Source: Hereditas. 2025 Aug 16;162:164. doi: 10.1186/s41065-025-00528-y (PMC12357469; doi:10.1186/s41065-025-00528-y)
Supplement: Supplementary file 3 — Supplementary Material 3 [file 41065_2025_528_MOESM3_ESM.docx]

eTable 3

| Structure | Algorithm | Models | Evaluation | Environment | Key aspects ofenhancing immunity | Refs |
| --- | --- | --- | --- | --- | --- | --- |
| Data Imputation | DCA | Autoencoder (AE) | DREMI | Keras,Tensorflow,scanpy | DCA effectively captures robust co-expression patterns among the top 500 genes utilizing either FCN or VAE architectures. It outperforms SAVER, scImpute, and MAGIC in gene clustering and the identification of differentially expressed genes. Additionally, it is integrated into the Scrubpy framework, which is designed for spatial transcriptomics analysis. | 1 |
|  | SAVER-X | AE+TL | t-SNE,ARI | R/sctransfer | SAVER-X is pretrained using cross-species expression data and decodes latent components to accurately infer true gene expression. It demonstrates robust performance across both samples and species. | 2 |
|  | DeepImpute | DNN | MSE,Pearson’scorrelation | Keras/Tensorflow | DeepImpute employs multiple shallow neural networks applied to gene subsets in parallel, significantly improving computational speed compared to methods like MAGIC and scImpute. It excels in identifying differential gene expression. | 3 |
|  | LATE | AE | MSE | Tensorflow | LATE is an unsupervised autoencoder that functions independently of external data, proving effective for highly sparse scRNA-seq data with numerous zero entries. | 4 |
|  | scGAMI | AE | NMI,ARI,HSandCS | Tensorflow | scGNN integrates graph structure with an autoencoder to capture higher-order relationships and impute missing values. It outperforms methods like MAGIC, SAVER, DCA, and scImpute, particularly on sparse datasets. | 5 |
|  | scIGANs | GAN | ARI,ACC,AUCandF-score | PyTorch | scIGANs utilize GANs to generate artificial expression profiles and model sparse expression patterns. They are more effective at distinguishing real from synthetic data compared to other methods and perform well on highly sparse datasets. | 6 |
| Batch Effect Correction | BERMUDA | AE+TL | KNNbatch-effecttest (kBET),theentropyof Mixing,SI | PyTorch | BERMUDA employs transfer learning to preserve unique batch characteristics while aligning shared structures. It effectively handles data with varying batch compositions. | 7 |
|  | DESC | AE | ARI,KL | Tensorflow | DESC reduces inter-batch variability through clustering, assuming it is smaller than biological variation. It manages batch-specific cell types and performs explicit clustering. | 8 |
|  | iMAP | AE+GAN | kBET,LocalInverse Simpson’sIndex(LISI) | PyTorch | MAP integrates autoencoders and GANs to progressively align batch differences through a multi-phase training process. It outperforms methods such as Harmony and scVI, particularly under complex batch conditions. | 9 |
| Clustering,latent representation,dimension reduction and data augmentation | Dhaka | VAE | ARI,SpearmanCorrelation | Keras/Tensorflow | Dhaka utilizes a 3D latent space to reduce dimensions and separate cell groups. It surpasses t-SNE, PCA, and scVI in clustering resolution. | 10 |
|  | scvis | VAE | KNNpreservation,log-likelihood | Tensorflow | scVI models technical noise and library size to learn a low-dimensional feature space, enabling clustering, batch correction, and expression analysis. It scales effectively to datasets with over one million cells. | 11 |
|  | scVAE | VAE | ARI | Tensorflow | scVAE is a variational autoencoder designed to capture gene variation in an embedded form. While it lags slightly behind scVI, it remains robust for regulatory network analysis. | 12 |
|  | VASC | VAE | NMI,ARI,HSandCS | H5py,keras | VASC employs a VAE-based nonlinear reduction method that outperforms t-SNE, PCA, and SIMLR in capturing developmental trajectories. | 13 |
|  | scDeepCluster | AE | ARI,NMI,clustering accuracy | Keras,Scanpy | scDeepCluster jointly learns latent representations and clustering labels, effectively capturing the diversity of cell types. | 14 |
|  | cscGAN | GAN | t-SNE,markergenes,MMD,AUC | Scipy,Tensorflow | cscGAN generates synthetic data for specific cell types to augment and balance datasets. It aids in classification tasks and simulates realistic data distributions. | 15 |
| Multi-functionalmodels (IM:imputation,BC:batchcorrection,CL:clustering) | scVI | VAE | IM:L1distance;CL:ARI, NMI,SI;BC:Entropyof Mixing | PyTorch,Anndata | scVI is a versatile framework that leverages variational inference for clustering, batch correction, differential expression analysis, and visualization. | 16 |
|  | LDVAE | VAE | Reconstructionerrors | PartofscVI | LDVAE is a lightweight variant of scVI that enhances the interpretation of latent variable features and aids in exploring gene relationships. | 17 |
|  | SAUCIE | AE | IM:R2statistics;CL:SI; BC: modifiedkBET; Visualization: Precision/Recall | Tensorflow | SAUCIE directly analyzes raw data for clustering, denoising, dimension reduction, batch correction, and anomaly detection. It is memory-efficient and fast, making it suitable for large-scale datasets. | 18 |
|  | scScope | AE | IM:Reconstructionerrors; BC:Entropyofmixing;CL: ARI | Tensorflow,Scikit-learn | scScope utilizes multi-layer networks for denoising and batch correction, performing well with sparse input data. It scales efficiently to datasets containing over 100k cells. | 19 |
| Cell-typeIdentification | Random Forest | Ensemble Tree | Accuracy, F1-score, ROC-AUC | scikit-learn | By integrating multiple decision trees, it becomes robust to noise and can effectively identify key gene features. This enhances the discrimination and understanding of immune states across complex cell types. | 20 |
|  | SVM | Kernel SVM | Accuracy, Precision, Recall, F1-score, ROC-AUC | scikit-learn, libSVM | It is well-suited for high-dimensional, small-sample data, offering clear decision boundaries and precise classification of cellular immune states. It supports multiple kernel functions, allowing flexible discrimination of different immune phenotypes. | 21 |
|  | DigitalDL Sorter | DNN | Pearsoncorrelation | R/Python/Keras | DigitalDLSorter utilizes a 4-layer deep neural network (DNN) to infer the proportions of cell types in bulk RNA-seq data, achieving a high correlation with the ground truth | 22 |
|  | scCapsNet | CapsNet | Cell-typePrediction accuracy | Keras,Tensorflow | scTAN combines attention mechanisms and autoencoders to capture complex expression dependencies, performing effectively in low-data scenarios. | 23 |
|  | netAE | VAE | Cell-type Prediction accuracy,t-SNE for visualization | pyTorch | netAE is a graph-based semi-supervised autoencoder that embeds cell relationships through local topology. It supports batch integration and trajectory modeling. | 24 |
|  | scDGN | DANN | Predicitonaccuracy | pyTorch | scDGN combines deep neural networks and graph structures to identify cell categories across different batches and conditions. It shows strong performance on public datasets. | 25 |

*References:*

| 1 | Eraslan G, Simon L M, Mircea M, et al. Single-cell RNA-seq denoising using a deep count autoencoder[J]. Nature communications, 2019, 10(1): 390. |  |
| --- | --- | --- |
| 2 | Wang J, Agarwal D, Huang M, et al. Data denoising with transfer learning in single-cell transcriptomics[J]. Nature methods, 2019, 16(9): 875-878. | |
| 3 | Arisdakessian C, Poirion O, Yunits B, et al. DeepImpute: an accurate, fast, and scalable deep neural network method to impute single-cell RNA-seq data[J]. Genome biology, 2019, 20(1): 211. | |
| 4 | Badsha M B, Li R, Liu B, et al. Imputation of single‐cell gene expression with an autoencoder neural network[J]. Quantitative Biology, 2020, 8(1): 78-94. | |
| 5 | Yu B, Chen C, Qi R, et al. scGMAI: a Gaussian mixture model for clustering single-cell RNA-Seq data based on deep autoencoder[J]. Briefings in bioinformatics, 2021, 22(4): bbaa316. | |
| 6 | Xu Y, Zhang Z, You L, et al. scIGANs: single-cell RNA-seq imputation using generative adversarial networks[J]. Nucleic acids research, 2020, 48(15): e85-e85. | |
| 7 | Wang T, Johnson T S, Shao W, et al. BERMUDA: a novel deep transfer learning method for single-cell RNA sequencing batch correction reveals hidden high-resolution cellular subtypes[J]. Genome biology, 2019, 20(1): 165. | |
| 8 | Li X, Wang K, Lyu Y, et al. Deep learning enables accurate clustering with batch effect removal in single-cell RNA-seq analysis[J]. Nature communications, 2020, 11(1): 2338. | |
| 9 | Wang D, Hou S, Zhang L, et al. iMAP: integration of multiple single-cell datasets by adversarial paired transfer networks[J]. Genome biology, 2021, 22(1): 63. | |
| 10 | Rashid S, Shah S, Bar-Joseph Z, et al. Dhaka: variational autoencoder for unmasking tumor heterogeneity from single cell genomic data[J]. Bioinformatics, 2021, 37(11): 1535-1543. | |
| 11 | Ding J, Condon A, Shah S P. Interpretable dimensionality reduction of single cell transcriptome data with deep generative models[J]. Nature communications, 2018, 9(1): 2002. | |
| 12 | Grønbech C H, Vording M F, Timshel P N, et al. scVAE: variational auto-encoders for single-cell gene expression data[J]. Bioinformatics, 2020, 36(16): 4415-4422. | |
| 13 | Wang D, Gu J. VASC: dimension reduction and visualization of single-cell RNA-seq data by deep variational autoencoder[J]. Genomics, proteomics & bioinformatics, 2018, 16(5): 320-331. | |
| 14 | Tian T, Wan J, Song Q, et al. Clustering single-cell RNA-seq data with a model-based deep learning approach[J]. Nature Machine Intelligence, 2019, 1(4): 191-198. | |
| 15 | Marouf M, Machart P, Bansal V, et al. Realistic in silico generation and augmentation of single-cell RNA-seq data using generative adversarial networks[J]. Nature communications, 2020, 11(1): 166. | |
| 16 | Lopez R, Regier J, Cole M B, et al. Deep generative modeling for single-cell transcriptomics[J]. Nature methods, 2018, 15(12): 1053-1058. | |
| 17 | Svensson V, Gayoso A, Yosef N, et al. Interpretable factor models of single-cell RNA-seq via variational autoencoders[J]. Bioinformatics, 2020, 36(11): 3418-3421. | |
| 18 | Amodio M, Van Dijk D, Srinivasan K, et al. Exploring single-cell data with deep multitasking neural networks[J]. Nature methods, 2019, 16(11): 1139-1145. | |
| 19 | Deng Y, Bao F, Dai Q, et al. Scalable analysis of cell-type composition from single-cell transcriptomics using deep recurrent learning[J]. Nature methods, 2019, 16(4): 311-314. | |
| 20 | Zhou Z, Ye C, Wang J, et al. Surface protein imputation from single cell transcriptomes by deep neural networks[J]. Nature communications, 2020, 11(1): 651. | |
| 21 | Zhou Y, Peng M, Yang B, et al. scDLC: a deep learning framework to classify large sample single-cell RNA-seq data[J]. BMC genomics, 2022, 23(1): 504. | |
| 22 | Torroja C, Sanchez-Cabo F. Digitaldlsorter: deep-learning on scRNA-Seq to deconvolute gene expression data[J]. Frontiers in Genetics, 2019, 10: 978. | |
| 23 | Wang L, Nie R, Yu Z, et al. An interpretable deep-learning architecture of capsule networks for identifying cell-type gene expression programs from single-cell RNA-sequencing data[J]. Nature Machine Intelligence, 2020, 2(11): 693-703. | |
| 24 | Dong Z, Alterovitz G. netAE: semi-supervised dimensionality reduction of single-cell RNA sequencing to facilitate cell labeling[J]. Bioinformatics, 2021, 37(1): 43-49. | |
| 25 | Ge S, Wang H, Alavi A, et al. Supervised adversarial alignment of single-cell RNA-seq data[J]. Journal of Computational Biology, 2021, 28(5): 501-513. | |
